# Influence of Repressive Histone and DNA Methylation upon D4Z4 Transcription in Non-Myogenic Cells

**DOI:** 10.1371/journal.pone.0160022

**Published:** 2016-07-28

**Authors:** Sunny Das, Brian P. Chadwick

**Affiliations:** Department of Biological Science, Florida State University, Tallahassee, Florida, United States of America; University of Minnesota, UNITED STATES

## Abstract

We looked at a disease-associated macrosatellite array D4Z4 and focused on epigenetic factors influencing its chromatin state outside of the disease-context. We used the HCT116 cell line that contains the non-canonical polyadenylation (poly-A) signal required to stabilize somatic transcripts of the human double homeobox gene *DUX4*, encoded from D4Z4. In HCT116, D4Z4 is packaged into constitutive heterochromatin, characterized by DNA methylation and histone H3 tri-methylation at lysine 9 (H3K9me3), resulting in low basal levels of D4Z4-derived transcripts. However, a double knockout (DKO) of DNA methyltransferase genes, *DNMT1* and *DNMT3B*, but not either alone, results in significant loss of DNA and H3K9 methylation. This is coupled with upregulation of transcript levels from the array, including *DUX4* isoforms (*DUX4-fl*) that are abnormally expressed in somatic muscle in the disease Facioscapulohumeral muscular dystrophy (FSHD) along with DUX4 protein, as indicated indirectly by upregulation of bondafide targets of DUX4 in DKO but not HCT116 cells. Results from treatment with a chemical inhibitor of histone methylation in HCT116 suggest that in the absence of DNA hypomethylation, H3K9me3 loss alone is sufficient to facilitate *DUX4-fl* transcription. Additionally, characterization of a cell line from a patient with Immunodeficiency, Centromeric instability and Facial anomalies syndrome 1 (ICF1) possessing a non-canonical poly-A signal and DNA hypomethylation at D4Z4 showed DUX4 target gene upregulation in the patient when compared to controls in spite of retention of H3K9me3. Taken together, these data suggest that both DNA methylation and H3K9me3 are determinants of D4Z4 silencing. Moreover, we show that in addition to testis, there is appreciable expression of spliced and polyadenylated D4Z4 derived transcripts that contain the complete *DUX4* open reading frame (ORF) along with DUX4 target gene expression in the thymus, suggesting that DUX4 may provide normal function in this somatic tissue.

## Introduction

Macrosatellite repeats (MSR) are composed of near identical repeat units arranged in tandem that can span anywhere from tens to thousands of kilobases (kb) in the human genome [1–3]. What biological purpose is served by the arrangement and maintenance of these large tandem repeats is unclear, given that many of these MSRs do not code for a functional gene product. While some MSRs, such as DXZ4 at Xq23 have regulatory roles such as mediating long-range interactions between regions of chromatin [4–5], others such as TAF11-Like MSR at 5p15.1 and D4Z4 at 4q35.2 have been shown to segregate with diseases such as schizophrenia [6] and onset of different types of FSHD (OMIM # 158900; 158901) [7–11], respectively. These instances clearly demonstrate the potential impact repeat elements can have on human health. Owing to its disease relevance, D4Z4 is one of the few MSRs that have been extensively characterized with substantial information on its genomic organization. However, no study has investigated how D4Z4 silencing is maintained in cell types outside of the context of the disease.

Two major variants of chromosome 4q35.2 exist, referred to as 4qA and 4qB, which differ primarily due to the presence of a unique 260 bp sequence located immediately distal to the D4Z4 MSR (referred to as pLAM) and a β–satellite repeat on a 4qA chromosome [9, 12–13]. Several different haplotypes exist within 4qA and 4qB that are distinguished by various sequence polymorphisms proximal and distal to the D4Z4 array [12, 14]. In addition to the 4q array, a highly homologous MSR resides in the subtelomeric region of chromosome 10q [15–16], along with numerous divergent D4Z4 monomers scattered throughout the genome on acrocentric chromosomes [17–18]. It is to be noted that only certain haplotypes within the 4qA variant chromosome are disease associated whereas 4qB and 10q haplotypes are non-pathogenic [10, 19–21]. Each monomer of the 4q D4Z4 array is composed of near identical copies of a 3.3 kb repeat unit that contain the ORF for double homeobox 4 (*DUX4*) protein [22–25]. Every D4Z4 monomer contains two exons, the first of which contains the ORF whereas the second contributes to the 3’ untranslated region (3’-UTR). In addition to the array encoded exons, additional unique exons located distal to the last repeat unit have been identified, including exons 3 through 7, in transcripts that are normally expressed in the germline [10, 26–27]. Alternative splicing involving exon 1 (with and without exon 2), and combinations of the downstream exons give rise to different *DUX4* transcript isoforms that are expressed in the germline or inappropriately in FSHD somatic tissues [27]. Importantly, exon-3 embedded within pLAM, contains a non-canonical poly-A signal (AUUAAA) that is unique to the 4qA haplotype [27]. This poly-A signal differs by a single-nucleotide from the canonical poly-A signal (AAUAAA) found in majority of human mRNA transcripts [28]. Transcripts consisting of exons 1–3 (herein referred to as *DUX4-fl*) are stabilized through polyadenylation and are pathogenic when expressed in adult somatic muscle, and inappropriately activate a germline-specific cascade of genes in somatic cells [29–32]. Exon 1 also contains a cryptic non-canonical splice donor site within the *DUX4* ORF that can be used to generate a short isoform (*DUX4-s*), coding for a non-pathogenic truncated form of DUX4 [26–27]. Additionally, transcripts expressed in the germline use a polyadenylation signal downstream of exon 7 [27]. It is to be noted that 10qA haplotypes also contain the pLAM region but they do not contain the ‘ATTAAA’ sequence and cannot generate stable *DUX4-fl* transcripts [10] using exons 1-2-3. Testis derived 10qA transcripts can use the exon 7 poly-A signal [27].

*DUX4* normally regulates germline-specific and early stem-cell development genes in the germline but is epigenetically repressed in somatic cells [29]. Given that *DUX4* is a double homeodomain transcription factor with a role in normal development and is expressed in the testis [27], it is important to study how transcription from the array is regulated in cells that have not been studied extensively due to lack of any discernable disease-associated phenotype in such non-myogenic cell types. Moreover, it is important to ask if D4Z4 is normally expressed in tissues other than testis, since such expression might allude to a putative normal function for DUX4 outside of the germline.

Our main goal for this study was to assess the contribution of repressive DNA and histone methylation in maintaining D4Z4 in a transcriptionally silent state in a non-muscle cell type that would be easily amenable to genetic and epigenetic manipulation. Additionally, we also asked if D4Z4 is normally transcribed in tissues other than testis and patient myotubes. We identified a non-myogenic cell line that carries a 4qA allele (hence the disease associated poly-A signal), and consequently has the potential to express polyadenylated *DUX4-fl* using exon-3 or exon-7. Therefore, these cells provided a platform to study how change to epigenetic modifications may trigger D4Z4 transcription outside of patient cells in which D4Z4 chromatin state is already altered and *DUX4-fl* is expressed.

## Materials and Methods

### Cell culture

HCT116, a male colon carcinoma cell line was obtained from the American Type Culture Collection (ATCC; No. CCL-247) (www.atcc.org), as was human embryonic kidney 293 cells (CRL-1573). HCT116 is characterized by a relatively stable, near diploid karyotype [33–35], and has been shown to be readily susceptible to genome engineering [36]. 1KO, 3BKO and DKO were obtained from Dr. Bert Vogelstein’s laboratory at Johns Hopkins University School of Medicine. EBV-transformed lymphoblast cell lines for FSHD1 (GM17939), ICF1 patient (GM08714) and her unaffected parents (GM08728 and GM0829) were all obtained from Coriell Cell Repositories at the Coriell Institute for Medical Research (www.coriell.org). hTERT-RPE1 (Human telomerase immortalized diploid female retinal epithelial cell line; C4000-1), hTERT-BJ1 (Human telomerase immortalized diploid male foreskin fibroblast cell line; C4001-1) and hTERT-HME1 (Human telomerase immortalized diploid female breast epithelial cell line; C4002-1) were purchased from Clontech Laboratories.

### Chaetocin treatment

0.5 X 10^6^ cells (for *DUX4* expression or BiS analyses) or 5 X 10^6^ cells (for ChIP) of HCT116, 1KO and 3BKO were initially seeded. 24 hours later, the media was replaced with fresh media supplemented with Chaetocin (Santa Cruz Biotechnology; Cat No. sc-200893), dissolved in DMSO at a final concentration of 400nM. Chaetocin supplemented media was changed every 24 hours for 2 days from first treatment. Cells were harvested 48 hours after first treatment, for subsequent experiments.

### Genotyping analysis

Genomic DNA was isolated from cells with the NucleoSpin Tissue kit (Machery-Nagel). An initial PCR was carried out to amplify a region of exon 3 of DUX4-fl using primers DUX4-Fwd-2 and DUX4-R-(DAS) (Table 1). The PCR products were then cloned into pDrive TA vector (Qiagen), before sequencing (Eurofins MWG Operon) and comparison to known consensus sequences for 4qA, 4qB and 10qA. Sequence alignments and comparisons were made using the Sequencher 5.0 (Gene Codes Corp.) software. Clones were compared to reference sequences for 4qA (Accession Number: FJ439133) and 10qA alleles (Accession Number: AL732375) [10].

**Table 1:** List of oligonucleotides.

Category	Primer Name	Primer Sequence (5’ to 3’)	Product Size
**Genotyping**	DUX4-Fwd-2	AGACCTGCGCGCAGTGCGCAC	226 bp
	DUX4-R-(DAS)	TGATCACACAAAAGATGCAAATC	
***DUX4-fl* RT-PCR (Nested)**	DUX4-3-F	CACTCCCCTGCGGCCTGCTGCTGGATGA	525 bp (with Intron 1) 381 bp (without Intron 1)
	DUX4-3-R	CCAGGAGATGTAACTCTAATCCAGGTTTGC	
	DUX4-1A-F	GAGCTCCTGGCGAGCCCGGAGTTTCTG	
	DUX4-184-R	GTAACTCTAATCCAGGTTTGCCTAGACAGC	
**D4Z4 qRT-PCR Exon 1**	DUX4-UTR-Fwd	AGGCGCAACCTCTCCTAGAAAC	114 bp
	DUX4-A-Rev	GCTCCTCCAGCAGAGCCCGGTATTC	
***DUX4-fl* qRT-PCR Exons 2–3**	DUX4-cDNA-F10	ACCGCGGAGAACTGCCATTC	159 bp
	DUX4-cDNA-R4	GACATTCAGCCAGAATTTCACG	
***DUX4* RT-PCR Exons 2–6 and Exons 2–7**	DUX4-Ex2-F1	GAGAGACTCCACACCGCGGA	178 bp for Exon 2–6 RT-PCR (germline transcript)
	DUX4-Ex6-R2	GAAACGTGGTATCTGGAGAG	
	DUX4-Ex7-R	CAGTAAGAGGACCTTGTGAG	201 bp for Exon 2–7 RT-PCR (germline transcript)
**BiS-D4Z4**	D4Z4-BiS-Fwd	AGGAAGGTAGGGAGGAAAAG	457 bp
	D4Z4-BiS-Rev	ACCCTTCCCTACATATTTCC	
**ChIP PCR**	D4Z4-Fwd-8	GGGACGCTGAGCGTTCCAG	277 bp
	D4Z4-Rev-7	GGACGCTGACCGTTTTCC	
**ChIP qPCR**	D4Z4-q-F1	CCGCGTCCGTCCGTGAAA	106 bp
	D4Z4-q-R1	TCCGTCGCCGTCCTCGTC	
***GAPDH* Control RT-PCR**	GAPDH-Fwd	GAAGGTGAAGGTCGGAGTC	226 bp
	GAPDH-Rev	GAAGATG GTGATGGGATTTC	
***GAPDH* Control qRT-PCR**	GAPDH-q-Fwd	CCCAATACGACCAAATCCGT	119 bp
	GAPDH-q-Rev	TCTCTGCTCCTCCTGTTCGA	
**Verification of HCT116 DNMT KOs Genomic DNA PCR**	DNMT1-Gen-F1	AAACTGGCAGGTGCTAACTG	251 bp
	DNMT1-Rev	AGATGTGATGGTGGTTTGCC	
	DNMT3B-Gen-F1	TTGGTTTTGCTCAGAGCCAG	227 bp
	DNMT3B-Gen-R1	ACGTGTGGGCAAGAGATTTC	
**Verification of HCT116 DNMT KOs RT-PCR**	DNMT1-Fwd	AATTATCCGAGGAGGGCTAC	293 bp
	DNMT1-Rev	AGATGTGATGGTGGTTTGCC	
	DNMT3B-Fwd	ATCAGAGGCCGAAGATCAAG	186 bp
	DNMT3B-Rev	ATTTCGAGTTCGGACAGCTG	
**DUX4 target gene expression RT-PCR**	TRIM43-Fwd	ACCCATCACTGGACTGGTGT	100 bp
	TRIM43-Rev	CACATCCTCAAAGAGCCTGA	
	MBD3L2-Fwd	CGTTCACCTCTTTTCCAAGC	142 bp
	MBD3L2-Rev	AGTCTCATGGGGAGAGCAGA	

### Chromatin immunoprecipitation (ChIP) and PCR analysis

Cells were fixed in culture media for 10 min at room temperature by addition of formaldehyde to 1% final concentration. Cross-linking was quenched for 5 min by addition of glycine to 125mM final concentration. Cells were washed and collected with ice-cold 1X PBS supplemented with 0.1 mg/ml 4-(2-Aminoethyl) benzenesulfonyl fluoride hydrochloride. Cells were resuspended at 7 X 10^6^ cells/ml in lysis buffer (1% SDS, 10mM EDTA, 50mM Tris, pH 8.0, containing 2 μg/ml Aprotinin, 2 μg/ml Leupeptin, 1 μg/ml Pepstatin and 0.1 μg/ml 4-(2-Aminoethyl) benzenesulfonyl fluoride hydrochloride). Sonication was performed with 0.2 ml of lysate per 1.7 ml tube for 20 cycles using a Bioruptor (Diagenode) set at high power, 30s on, 30s off cycle. The bath was allowed to cool after every five cycles. Lysate was pre-cleared with protein-A agarose beads, before addition of primary antibody and incubation overnight at 4°C. Immune complexes were collected the next day with protein-A agarose and washed at 4°C twice for 5 min each with low wash buffer (0.1% SDS, 1% Triton X-100, 2mM EDTA, 150mM NaCl, 20mM Tris, pH 8.0), once for 5 min with high wash buffer (0.1% SDS, 1% Triton X-100, 500mM NaCl, 20mM Tris, 8.0) and once for 5 min with TE buffer (10mM Tris, pH 8.0, 1mM EDTA). Protease inhibitors were used at the same concentration as in the lysis buffer. Chromatin was eluted at room temperature with 100mM NaHCO3, 1% SDS and cross-links reversed overnight at 65°C after addition of NaCl to 0.2 M. Residual RNA was removed for 30 min at 37°C with RNase A, then protein by a 120-min incubation at 42°C with proteinase K (7.2 mUnits). DNA was purified with the QIAquick PCR purification kit (Qiagen). Protein-A agarose, RNase A, proteinase K and all protease inhibitors were obtained from Roche Applied Science. DNA immunoprecipitated (IP samples) with either rabbit polyclonal anti-H3K4me2 antibody (Active Motif; Cat # 39141), rabbit polyclonal anti-H3K9me3 (Active Motif; Cat # 39161) or rabbit serum (RS; negative control) was assessed by either qualitative PCR with D4Z4-Fwd-8 and D4Z4-Rev-7 or qPCR with Q-PCR-Fwd and Q-PCR-Rev (Table 1). Chromatin controls not treated with any antibody was labeled as Input sample. Standard PCR was carried out with HotStar Taq Plus (Qiagen) using the following thermocycling conditions: initial denaturation of 5 min at 94°C, followed by 40 cycles of 30s at 94°C, 30s at 58°C, 30s at 72°C along with a final extension step of 10 min at 72°C. Amplified products were examined on 2% agarose (Agarose Unlimited) gels in 1 X TAE. qChIP was performed on a CFX96 (Biorad, Hercules, CA, USA) using EvaGreen 2X qPCR Mastermix (ABM; Cat No. Mastermix-S). The two-step amplification cycle began with a 10 min incubation at 95°C, followed by 40 cycles of 15s at 95°C and 1 min at 62°C. Fluorescence was measured at the end of each of the 40 cycles. Afterwards, a melt curve was generated to confirm the absence of primer-dimer by measuring the fluorescence after heating for 5s in 0.5°C increments, beginning at 65°C and ending at 95°C. Enrichment of H3K9me3 or H3K4me2 in qChIP results was calculated after adjusting for input dilution, RS background and subsequently expressing corrected IP values as percentage of input. Triplicates were used for each sample.

### Bisulfite modification of genomic DNA, cloning and sequencing analysis of CpG methylation

Genomic DNA was isolated from cells with the NucleoSpin Tissue kit (Machery-Nagel). Unmethylated cytosines were converted to uracil with the EpiTect Bisulfite conversion and cleanup kit (Qiagen). Bisulfite modified DNA was used as a template for PCR with HotStar Taq Plus (Qiagen) and the primers D4Z4- BiS-Fwd and D4Z4-BiS-Rev (Table 1). PCR products were cloned into pDrive TA vector (Qiagen), and sequenced (Eurofins MWG Operon). CpG methylation was calculated based on a pairwise alignment of sequenced clones to an in silico prepared fully methylated reference sequence using the Bisulfite Sequencing DNA Methylation Analysis (BISMA) software (http://services.ibc.uni-stuttgart.de/BDPC/BISMA/) [37]. The number of methylated CpG sites was expressed as a percentage of the total number of CpG in each clone. Values from all clones were averaged to assign percentage methylation for each sample.

### Isolation of RNA and preparation of cDNA

Total RNA was isolated from cells using the NucleoSpin RNA II kit (Machery-Nagel). Total RNA (1ug/ul stock) for all tissues was purchased from Clontech Laboratories or Agilent Technology (S1 Table). First-strand cDNA was prepared from equal amounts of starting RNA (2ug total RNA) with either oligo-dT primers or random hexamers, with and without M-MuLV reverse transcriptase (RT) according to the manufacturer’s instructions (NEB). cDNAs prepared with and without RT were used as templates for both qualitative and quantitative PCR. cDNA was diluted to half of original concentration prior to qualitative RT-PCR and qRT-PCR (dilution one-sixth of original concentration for cDNA made with random hexamers). Both RT-PCR and qRT-PCR reactions were carried out with equal starting amounts of cDNA for each sample.

Genomic PCR and RT-PCR for verification of identity of HCT116 DNMT KOs

Genomic DNA isolated from HCT116, 1KO, 3BKO and DKO was amplified using primers DNMT1-Gen-F1 and DNMT1-Rev located within *DNMT1* and DNMT3B-Gen-F1 and DNMT3B-Gen-R1 located within *DNMT3B* (Table 1). For RT-PCR the primers used were DNMT1-Fwd and DNMT1-Rev for *DNMT1* and DNMT3-Fwd and DNMT3-Rev for *DNMT3B*. For RT-PCR, *GAPDH* expression was also assessed as a positive control using primers GAPDH-Fwd and GAPDH-Rev (Table 1). Both genomic and RT-PCR was carried out using HotStar Taq Plus (Qiagen) using the following thermocycling conditions: initial denaturation of 5 min at 94°C, followed by 40 cycles of 30s at 94°C, 30s at 58°C, 30s at 72°C along with a final extension step of 10 min at 72°C. Amplified products were examined on 2% agarose (Agarose Unlimited) gels in 1X TAE.

### RT-PCR and qRT-PCR for DUX4 expression analysis

*DUX4-fl* expression was detected using a nested PCR strategy that will selectively amplify transcripts containing the 3’ end of exon 1 and the region of exon 3 containing the non-canonical polyadenylation signal, using a first set of primers DUX4-3-F and DUX4-3-R followed by nesting with a second set DUX4-1A-F and DUX4-184-R [30] (Table 1). Thermocycling was carried out using Phusion polymerase (NEB) with a compatible Phusion 5 X GC Buffer and DMSO (3%) with an initial denaturation step of 30s at 98°C, followed by either 12 cycles (first set of nested primers) or 40 cycles (second set of nested primers) of 10s at 98°C, 30s at 58°C, 15s at 72°C along with a final extension step of 10 min at 72°C. Amplified products from the first PCR were cleaned using the MinElute PCR purification Kit (Qiagen) and used for the second round of PCR. Final amplified products were examined on 1.5% agarose (Agarose Unlimited) gels in 1X TAE for the above. For analyzing expression of polyadenylated transcripts utilizing exon 7 poly-A, RT-PCR was carried out using forward primer DUX4-Ex2-F1 (in exon 2) and two independent reverse primers DUX4-Ex6-R2 (in exon 6) or DUX4-Ex7-R (in exon 7). Thermocycling was carried as above using Phusion polymerase with 5X GC Buffer without DMSO with same conditions as above for 35 cycles and run on a 2% agarose gel. The PCR products were cloned into pMiniT (NEB) and inserts sequenced (Eurofins Genomics). The sequences of these clones were submitted to Genbank (Accession numbers KX467569 and KX467570). For all the above RT-PCRs, *GAPDH* expression was also assessed as a control using primers GAPDH-Fwd and GAPDH-Rev (Table 1) with HotStar Taq Plus (Qiagen) using the above thermocycling conditions. Final amplified products were examined on 2% agarose (Agarose Unlimited) gels in 1X TAE for the above.

qRT-PCR for *DUX4* transcripts was carried out using two independent sets of primers. While a combination of DUX4-UTR-Fwd and DUX4-A-Rev amplified any transcript containing the 3’ end of exon 1 of *DUX4*, the combination of DUX4-cDNA-F10 and DUX4-cDNA-R4 (Table 1) amplified transcripts containing exons 2 and 3. qGAPDH-Fwd and qGAPDH-Rev (Table 1) were used as a control for normalization during analysis of results. qRT-PCR was performed with the same reagents and conditions as for qChIP. *DUX4* expression for each sample was analyzed relative to *GAPDH* expression after correcting for background noise from–RT samples, using the ΔΔCt method. Triplicates were used for each sample.

### qRT-PCR for DUX4 target gene expression analysis

TRIM43 expression levels were assessed using primers TRIM43-Fwd and TRIM-Rev while MBD3L2 expression levels were assessed using primers MBD3L2-Fwd and MBD3L2-Rev (Table 1) using oligo-dT primed cDNA of each sample. qGAPDH-Fwd and qGAPDH-Rev were used as a control for normalization during analysis of results. qRT-PCR was performed with the same reagents and conditions as for qChIP. Expression for each sample was analyzed relative to that of *GAPDH* after correcting for background noise from–RT samples, using the ΔΔCt method. Triplicates were used for each sample.

### Oligonucleotides

All oligonucleotides were synthesized using the service of Eurofins Genomics. Oligonucleotide sequences and their applications are listed in Table 1 and S2 Fig.

### Statistical analysis

D4Z4 CpG BiS: Average CpG methylation values (percentage) from independent clones were subsequently used to determine statistically significant differences between pairs of samples using a paired two-tailed student’s t-test.

H3K9me3/H3K4me2 qChIP: Effective enrichment (IP–RS) values (normalized to and expressed as percentage of input) were used to determine statistically significant differences between pairs of samples using a paired two-tailed student’s t-test.

D4Z4/*DUX4-fl* qRT-PCR: Expression values were subsequently used to determine statistically significant differences between pairs of samples using a paired two-tailed student’s t-test.

## Results

### D4Z4 is packaged into constitutive heterochromatin in HCT116 cells, which possess 4qA allele

**Fig 1:**
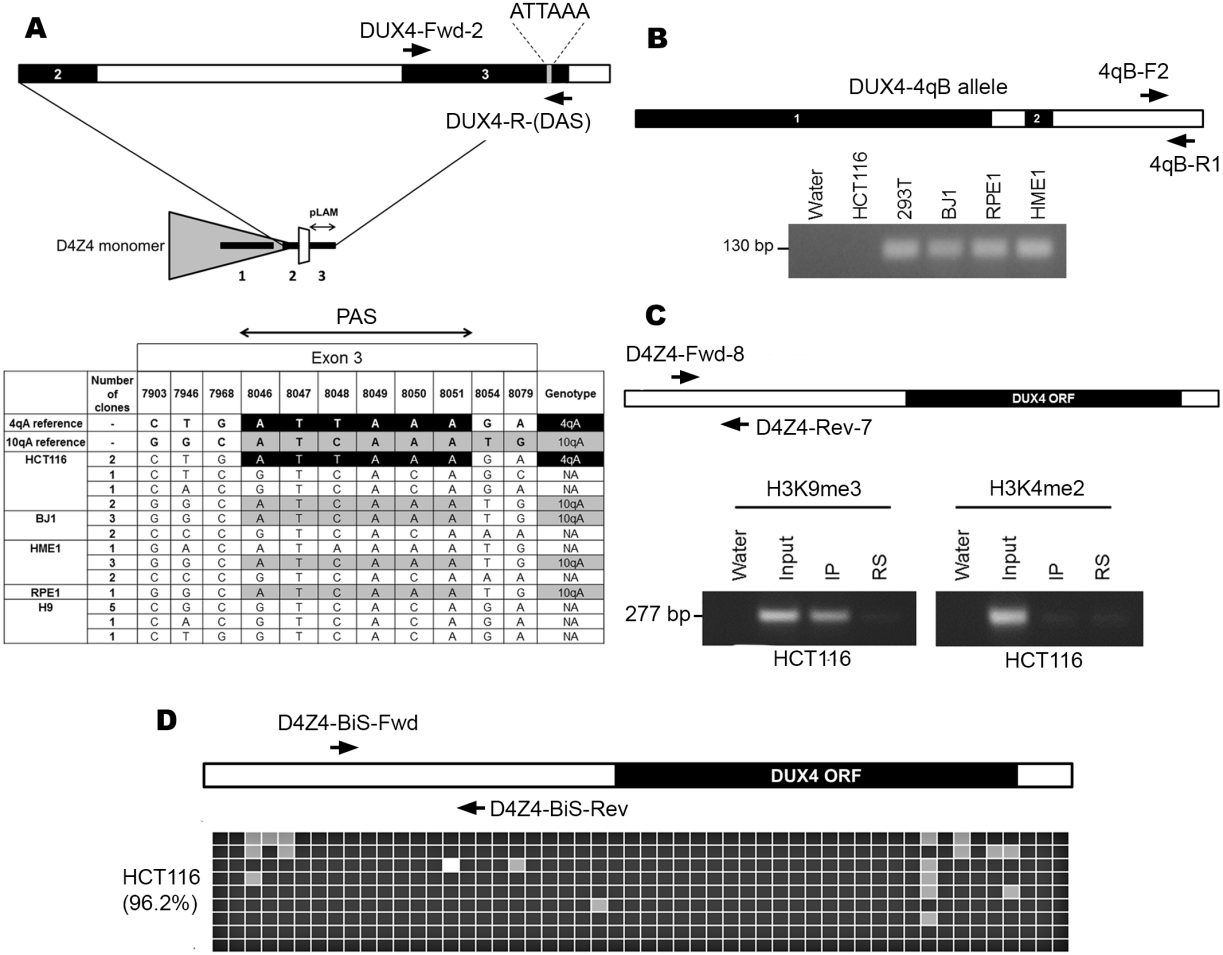
Genotyping and characterization of HCT116 at D4Z4. (A) Top panel depicts the primer map for genotyping the poly-A signal that stabilizes *DUX4* transcripts. A single D4Z4 monomer (grey triangle) containing exons 1, 2 and 3 (thick black lines) of DUX4, along with the pLAM region is shown below, with the region spanning exons 2 and 3 (interrupted by Intron 2 in white) expanded on top. Arrows indicate location of primers for genotyping PCR. Genotyping results in the bottom panel indicate cell lines surveyed and number of clones observed for each haplotype. The numbers on top indicate the positions in base pairs that correspond to single nucleotide polymorphisms (SNPs) characteristic of 10qA and permissive 4qA alleles in this region based on Accession Numbers AL732375 and FJ439133, respectively. The reference permissive 4qA allele containing poly-A signal ATTAAA (black) and 10qA (grey) are indicated (first two rows) and matches are represented likewise for the samples. Non-matching sequences are annotated as not assigned (NA). (B) Labeled arrows show location of primers at the distal edge of D4Z4 that were used to detect 4qB alleles. A representative image of an ethidium bromide–stained agarose gel showing PCR results is depicted below with cell lines indicated above. Product size indicated on the left. (C) Labeled arrows show location of primers, relative to the *DUX4* ORF (black rectangle) within each D4Z4 monomer (open rectangle) for PCR of ChIP samples. Immediately below is a representative image of ethidium bromide–stained agarose gels showing PCR results for HCT116 ChIP with anti-H3K9me3 (left) and anti-H3K4me2 (right). Product size is indicated on the left. Samples include water, input, ChIP elution (IP), and a rabbit serum (RS) control. (D) Labeled arrows show location of primers, relative to the DUX4 ORF (black rectangle) within each D4Z4 monomer (open rectangle). Result of bisulfite analysis for 52 CpG sites in HCT116 (average percentage methylation value shown on the left within brackets) within D4Z4 is shown below. Methylated cytosines are represented by black squares whereas unmethylated ones are colored grey. DNA variants that result in a sequence that is no longer a CpG are colored white. Each row of squares represents DNA sequence obtained from an independent single clone.

DUX4 containing D4Z4 transcripts can be stabilized by polyadenylation using the canonical polyA signal associated with exon-7, as seen in the germline [27], or for those cells that possess a 4qA haplotype on a chromosome 4, the non-canonical polyadenylation signal AUUAAA (ATTAAA in genomic DNA) found in exon-3 that is unique to 4qA variants can potentially be used [10]. We genotyped a panel of cell lines by PCR across exon-3 of *DUX4*, followed by sequencing of cloned PCR products, in order to identify those cell lines that possess this 4qA-associated variant. Among the cell lines surveyed, we found that HCT116 (a male colon carcinoma epithelial cell line that has been reported to be near diploid with a relatively stable chromosome karyotype [33–35], contained matches to the permissive 4qA allele and to the 10qA allele (Fig 1A). We also assessed the cell lines for the presence of a 4qB allele. PCR was performed using primers capable of specifically amplifying a DNA fragment unique to 4qB haplotypes. This analysis revealed that HCT116 is 4qB negative and therefore is likely 4qA homozygous (Fig 1B). Although we did not determine whether the *DUX4* poly-A is present in both 4qA alleles of this cell line, the presence of at least one poly-A signal is sufficient to stabilize *DUX4* transcripts that include exon-3.

HCT116 has previously been shown to have an altered chromatin state at the unrelated X-linked MSR DXZ4 [38]. Therefore, we first sought to determine if D4Z4 is still packaged into constitutive heterochromatin. To assess this, we initially performed ChIP using antibodies against the heterochromatic marker H3K9me3 and the euchromatic marker H3K4me2, followed by PCR using primers that amplify within each D4Z4 monomer. H3K9me3, but not H3K4me2, was readily detected (Fig 1C). Next, we assessed DNA methylation levels at D4Z4 by amplifying a DNA fragment containing 52 CpG sites from a bisulfite-treated template. The interval in question corresponds to a sequence located upstream of the *DUX4* ORF in each D4Z4 monomer. We observed that HCT116 is methylated (96.2% of CpGs) at D4Z4 (Fig 1D). Taken together, these results show that HCT116 has at least one *DUX4*-stabilizing poly-A signal and that D4Z4 is packaged into constitutive heterochromatin. Therefore, we selected this cell line to investigate how *DUX4* expression might be influenced by perturbation of CpG and H3K9 methylation.

### Combined loss of DNA methyltransferase genes *DNMT1* and *DNMT3B*, compromises heterochromatin at D4Z4

**Fig 2:**
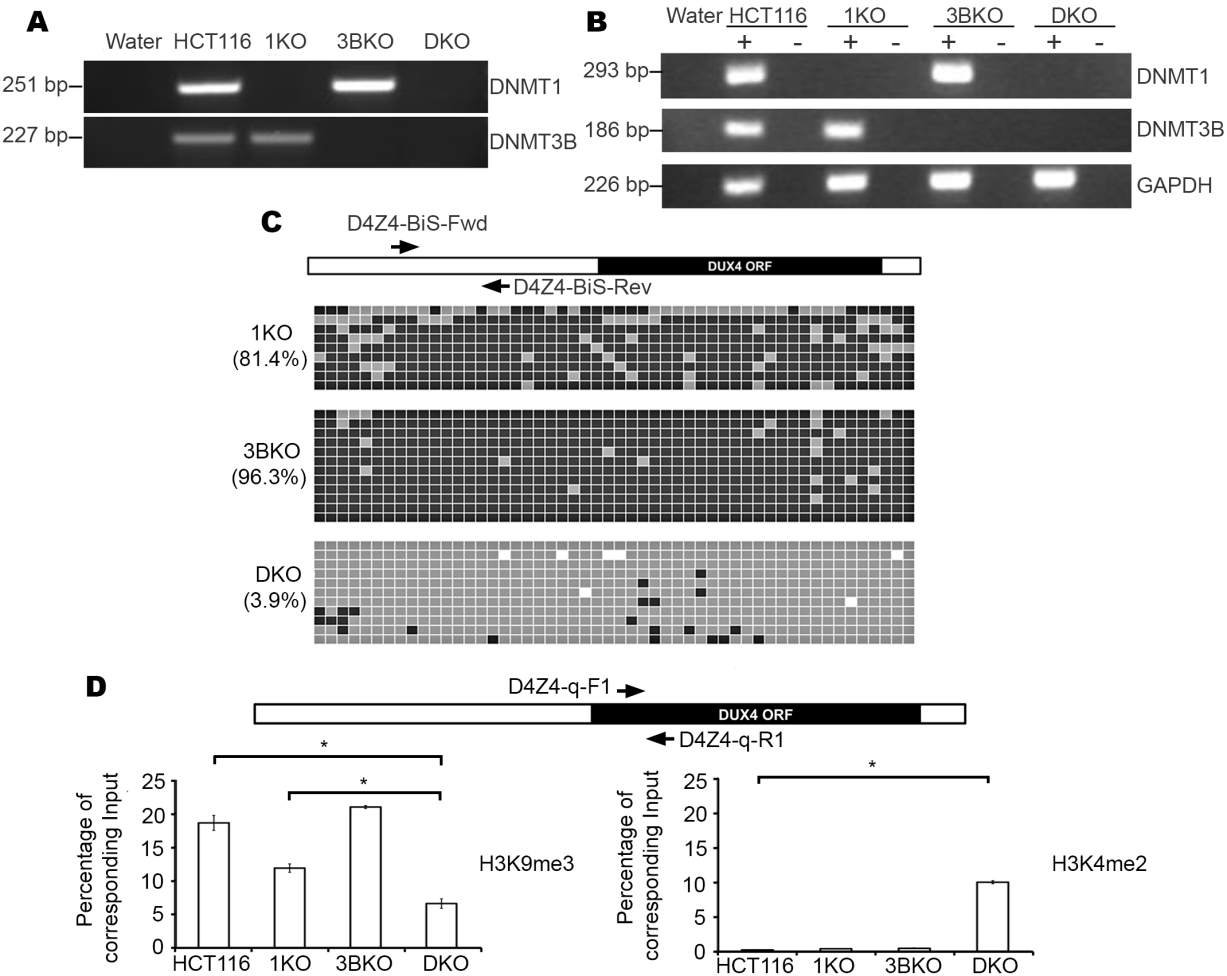
Identification and characterization of DNMT knockouts of HCT116. (A) Ethidium bromide–stained agarose gel images showing genomic PCR validation of parental HCT116 and its DNMT KO cell lines based on presence or absence of products for *DNMT1* and *DNMT3B*. (B) Ethidium bromide–stained agarose gels showing RT-PCR results from cDNA samples with (+) and without (-) reverse transcriptase for detecting *DNMT1* and *DNMT3B*. Respective *GAPDH* controls are shown at the bottom. (C) Primer map and results of bisulfite analysis for 1KO, 3BKO and DKO cell lines displayed in the same manner as described in Fig 1D. (D) Results of qPCR on HCT116, 1KO, 3BKO and DKO ChIP at D4Z4 for H3K9me3 and H3K4me2. Labeled arrows show location of primers, relative to the DUX4 ORF (black rectangle) within each D4Z4 monomer (open rectangle) for qPCR of ChIP samples. Sample names are indicated on the X-axis while enrichment values on the Y-axis are expressed as percentage of corresponding input samples, after normalization with respect to corresponding RS samples. All values are obtained by averaging results from triplicates for each sample. Error bars represent standard error (n = 3). Statistical significance is indicated (* indicates p = < 0.001).

HCT116 is readily targeted by homologous recombination [36], as demonstrated by the isolation of various clonal lines that have had specific genes disrupted, including knockouts/hypomorphs of the DNA methyltransferase genes *DNMT1* (1KO), *DNMT3B* (3BKO) and a double-knockout line (DKO) with both *DNMT1* and *DNMT3B* disrupted [39–40]. 1KO was generated by targeted deletion of exons 3, 4 and 5 of the *DNMT1* gene, 3BKO by targeted deletion of exons 2 through 21 of *DNMT3B*, whereas both genes are disrupted in DKO. The deletion in *DNMT1* produces a hypomorphic truncated-isoform of the DNMT1 protein that lack parts of the regulatory N-terminal domain but contains the catalytic C-terminal domain in both 1KO and DKO [41]. We first validated the identity of these cell lines after obtaining them. In order to do this we designed different primer sets to deleted regions of *DNMT1* and *DNMT3B* and performed both genomic DNA PCR and reverse transcription PCR (RT-PCR) with HCT116, 1KO, 3BKO and DKO genomic DNA (Fig 2A) and cDNA (Fig 2B), respectively. As expected, HCT116 was positive for both *DNMT1* and *DNMT3B*, 1KO was negative for *DNMT1* but positive for *DNMT3B*, 3BKO was negative for *DNMT3B* but positive for *DNMT1* whereas DKO was negative for both *DNMT1* and *DNMT3B*.

Given the loss of DNMT genes in these knockouts, we sought to determine CpG methylation at D4Z4 by bisulfite sequence (BiS) analysis (Fig 2C) as was done for parental cells (Fig 1D). While loss of *DNMT3B* alone does not noticeably impact D4Z4 methylation in 3BKO (96.3% compared to 96.2% in parental HCT116), there is significant hypomethylation in the *DNMT1* hypomorph, 1KO (81.4%, p = <0.05), and very significant methylation loss in DKO compared to parental cells (3.9% compared to 96.2%, p = <0.001) (Fig 2C).

Given the drastic reduction in CpG methylation in DKO compared to HCT116, we next sought to determine if histone methylation at D4Z4 is affected in the knockouts. In order to do this we carried out qPCR with primers within each D4Z4 monomer to amplify ChIP samples from HCT116, 1KO, 3BKO and DKO, immunoprecipitated with anti-H3K9me3 or anti-H3K4me2 antibodies. This revealed that in comparison to parental HCT116 cells, H3K9me3 is significantly reduced in DKO but not in 1KO or 3BKO (Fig 2D; left panel). Additionally, H3K4me2 levels are significantly increased DKO but not in 1KO or 3BKO (Fig 2D; right panel). Taken together, these observations indicate a substantial loss of constitutive heterochromatin and switch toward a more euchromatic configuration in DKO.

### *DUX4* isoforms implicated in FSHD are reactivated in the HCT116 DNMT double knockout

**Fig 3:**
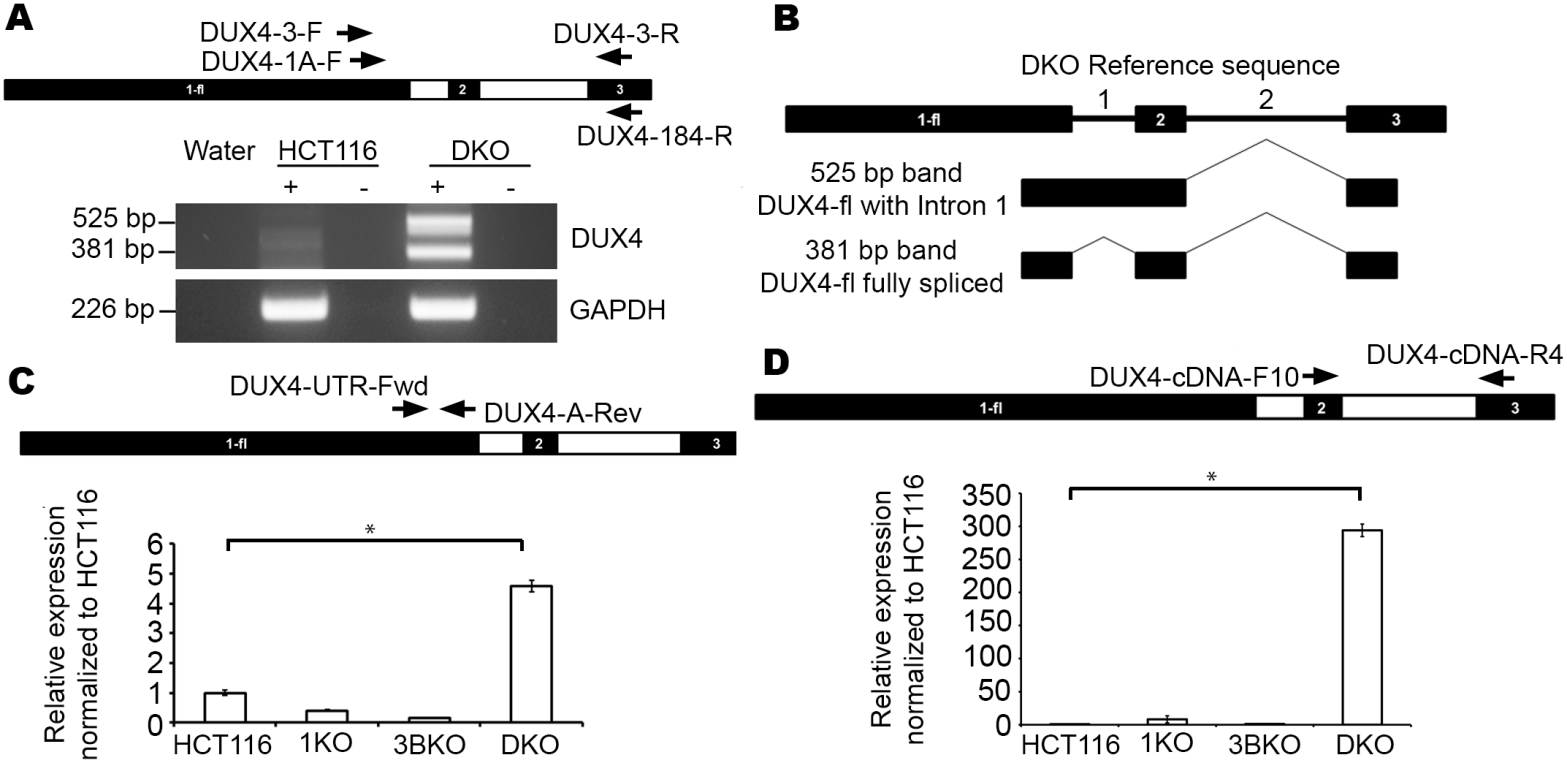
D4Z4/*DUX4-fl* transcription in HCT116 DNMT knockouts. (A) Labeled arrows show location of nested primer sets, relative to the most distal D4Z4 monomer (exons 1 and 2 in black rectangles) and the immediately downstream pLAM region containing exon 3 (black rectangle) for detection of *DUX4-fl* transcripts. Representative image of ethidium bromide–stained agarose gels showing PCR results are depicted below with cell lines indicated on top treated with (+) and without (-) reverse transcriptase along with water control. Expected product sizes are indicated on the left. A *GAPDH* amplification positive control for all samples is shown at the bottom. The two bands in *DUX4* panel correspond to amplified full length isoforms with (525 bp) and without (381 bp) intron 1. (B) Schematic representation of *DUX4-fl* isoforms transcribed in DKO. Also refer to S1 Fig (C) Labeled arrows show location of primers at 3’ end of exon 1, relative to the most distal D4Z4 monomer for detection of transcripts with the full *DUX4* ORF by qRT-PCR with cDNA samples made with random hexamers. Below it are results of qRT-PCR for D4Z4 transcription in HCT116 and its DNMT knockouts (X-axis) and expression levels with respect to HCT116 (arbitrarily set at 1; Y-axis). All values are obtained by averaging results from triplicates for each sample. Error bars represent standard error (n = 3). Statistical significance is indicated (* p = < 0.05). (D) Labeled arrows show location of primers, relative to the most distal D4Z4 monomer for detection of spliced and polyadenylated *DUX4* transcripts by qRT-PCR with cDNA samples made with oligo-dT primers. Below it are results of qRT-PCR for *DUX4-fl* in HCT116 and its DNMT knockouts (X-axis) and expression levels with respect to HCT116 (arbitrarily set at 1; Y-axis). All values are obtained by averaging results from triplicates for each sample. Error bars represent standard error (n = 3). Statistical significance is indicated (* p = < 0.001).

Since we observed a dramatic shift to a more euchromatic configuration in DKO cells at D4Z4, we next explored the possibility of *DUX4* mRNA expression in this cell line. If expressed, such transcripts should be stable due to presence of the *DUX4*-stabilizing poly-A signal. Given that D4Z4 is a GC-rich repeat and *DUX4* shows very low-level stochastic expression [13,27,42], we used a nested RT-PCR strategy with primers previously described in literature [27] that would exclusively pick up the *DUX4-fl* transcript (Fig 3A). We found that DKO indeed expresses both disease-associated isoforms of *DUX4-fl* transcript while HCT116 does not express either of them. Additionally, we TA cloned and sequenced each of these PCR products from DKO and aligned these sequences to annotated transcripts reported on the UCSC Genome browser (www.genome.ucsc.edu) [43] to verify the nature and identity of these transcripts (Fig 3B). As expected, we found that the smaller product (381 bp) corresponds to the fully spliced *DUX4-fl* transcript that has spliced out Introns 1 and 2. Notably, Intron 1 starts with a non-canonical 5’-GA splice donor sequence instead of the conventional 5’-GT, which is observed in almost 99% of all splice donor sites [44–45]. In DKO, the larger (525 bp) band is the *DUX4-fl* isoform that retains Intron 1, potentially reflecting less efficient splicing of this non-conventional intron-exon boundary. Nevertheless, the *DUX4* ORF is not affected by the presence or absence of this intron and therefore neither isoform impacts the coding potential of the transcript. As a positive control, expression of *DUX4-fl* isoforms was also observed in cDNA prepared from a FSHD1 patient lymphoblast cell line (GM17939; data not shown). We were unable to detect *DUX4-fl* by this strategy in 1KO and 3BKO cells (data not shown).

In order to quantify *DUX4* mRNA expression levels in DKO, we performed qRT-PCR. We analyzed transcription levels in HCT116, 1KO, 3BKO and DKO. A testis sample, derived from pooled testis tissue from multiple unrelated males (See S1 Table) and an FSHD lymphoblast (GM17939) were used as a positive controls (data not shown). Two sets of primers were determined to be suitable for qRT-PCR.

The first set is specific to exon 1. Therefore, they will amplify general transcription from any monomer in the D4Z4 array from both 4q and 10q in cDNA synthesized using random hexamers. We will call this “D4Z4 transcription” to differentiate it from exclusive spliced *DUX4-fl* transcription that includes exon 3. Very low levels of transcript were detected in HCT116, 1KO and 3BKO, whereas a significant increase was detected in DKO (Fig 3C).

The second primer set is located in exons 2 (forward primer) and 3 (reverse primer). It is possible that the reverse primer (in exon 3) also anneals to the same sequence at chromosome 10qA. However, since for this analysis we generated cDNA by selecting for polyadenylated mRNA, all transcripts amplified by qRT-PCR likely arise from 4qA (10qA transcripts would be unstable owing to lack of ‘ATTAAA’) i.e. spliced and polyadenylated *DUX4*. Similar to D4Z4 transcription, HCT116, 1KO and 3BKO detected very low levels of *DUX4-fl*, whereas there is a significant increase in DKO (Fig 3D). Similar results were obtained in qRT-PCR with cDNA samples made with random hexamers (data not shown). The difference in fold-change expression labels between the two primer sets could be attributed to the fact that the exon 1 primer set detects general D4Z4 transcription in random-primed cDNA whereas the exon 2–3 primer set detects spliced polyadenylated *DUX4* that could also amplify both *DUX4-s* and *DUX4-fl* transcripts.

DNA demethylation is known to activate myogenic pathways in non-myogenic cell types [46–48]. Moreover, DUX4 protein expression is known to be induced by myogenic factors [49]. Since DKO has undergone DNA and/or histone methylation changes and also showed an upregulation of D4Z4/*DUX4-fl* transcripts, we questioned if genes involved in myogenic pathways are activated in these cells. We found that Myogenic Differentiation 1 (*MYOD1*) and Myosin, Heavy Chain 2, Skeletal Muscle, Adult (*MYH2*) genes were upregulated only in DKO cells but not in HCT116 or the single DNMT KOs (S2A Fig).

### DUX4 target genes are upregulated in DKO

**Fig 4:**
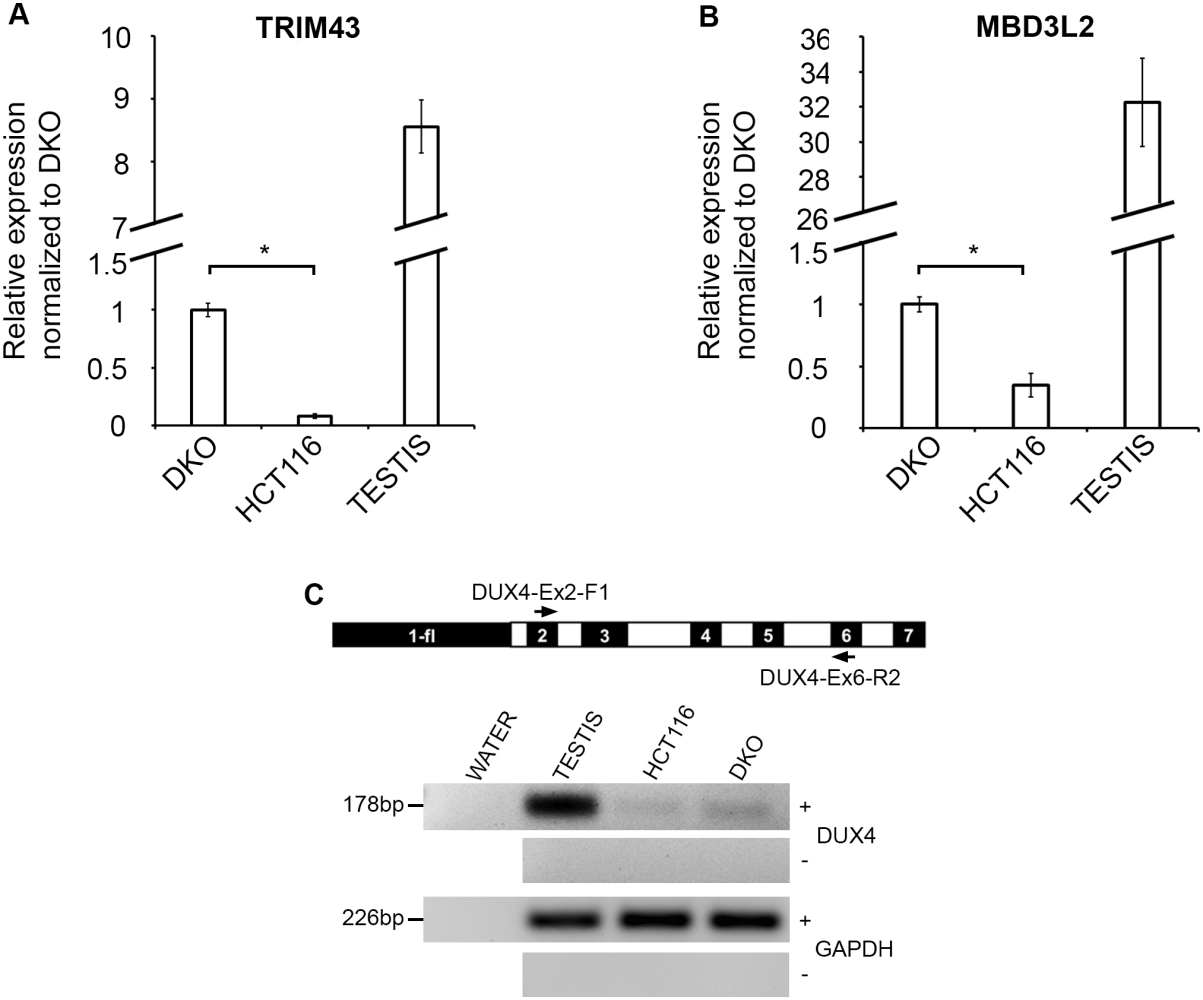
DUX4 target gene expression in HCT116 and DKO. (A) Results of qRT-PCR for DUX4 target genes TRIM43 in the HCT116, DKO and Testis (X-axis) expressed as fold change relative to expression in DKO (arbitrarily set at 1; Y-axis), normalized with respect to GAPDH expression. All values are obtained by averaging results from triplicates for each sample. Error bars represent standard error (n = 3). Statistical significance is indicated (* p = < 0.001). (B) Results of qRT-PCR for DUX4 target genes MBD3L2 in the HCT116, DKO and Testis (X-axis) expressed as fold change relative to expression in DKO (arbitrarily set at 1; Y-axis), normalized with respect to GAPDH expression. All values are obtained by averaging results from triplicates for each sample. Error bars represent standard error (n = 3). Statistical significance is indicated (* p = < 0.05). (C) Primer map and representative image of ethidium bromide–stained agarose gels showing RT-PCR results showing expression of non-pathogenic polyadenylated mRNA transcripts. Cell lines indicated on top treated with (+) and without (-) reverse transcriptase along with water control and testis as a positive control. Expected product sizes are indicated on the left. A *GAPDH* amplification positive control for all samples is shown at the bottom.

Although our study focuses on chromatin states at D4Z4 and its effect on transcript levels, in order to verify if transcripts arising in cell types other that muscle results in DUX4 protein we assessed protein levels in HCT116 and DKO using indirect immunofluorescence (IF) and Western Blotting (WB) with two different monoclonal antibodies raised against the DUX4 C-terminal domain [50], the results of which were inconclusive (data not shown). Hence, we quantified transcription of two robust downstream DUX4 targets [31] *TRIM43* and *MBD3L2*, with previously published primers [31] in HCT116 and DKO, along with a testis sample as a positive control. We found that both these genes are upregulated in DKO compared to HCT116 (Fig 4A and 4B), suggesting that DUX4 protein levels are higher in DKO. Given the fact that HCT116 did not express high levels of DUX4 transcripts spliced from Exon 3 (Fig 3D) yet seemed to generate some protein, we questioned if the protein was derived from DUX4 transcripts utilizing any downstream poly-A signal. Alternatively spliced transcripts in the germline are known to contain exons 1-2-6-7 [27], utilizing the poly-A signal downstream of exon 7. We thus performed RT-PCR on the HCT116 and DKO with testis as a positive control using forward and reverse primers in exons 2 and 6, respectively and found expression of transcripts of the expected size at low levels in HCT116 and higher levels in DKO (Fig 4C).

### Severe D4Z4 hypomethylation alone on a permissive chromosome may be sufficient for *DUX4* reactivation

**Fig 5:**
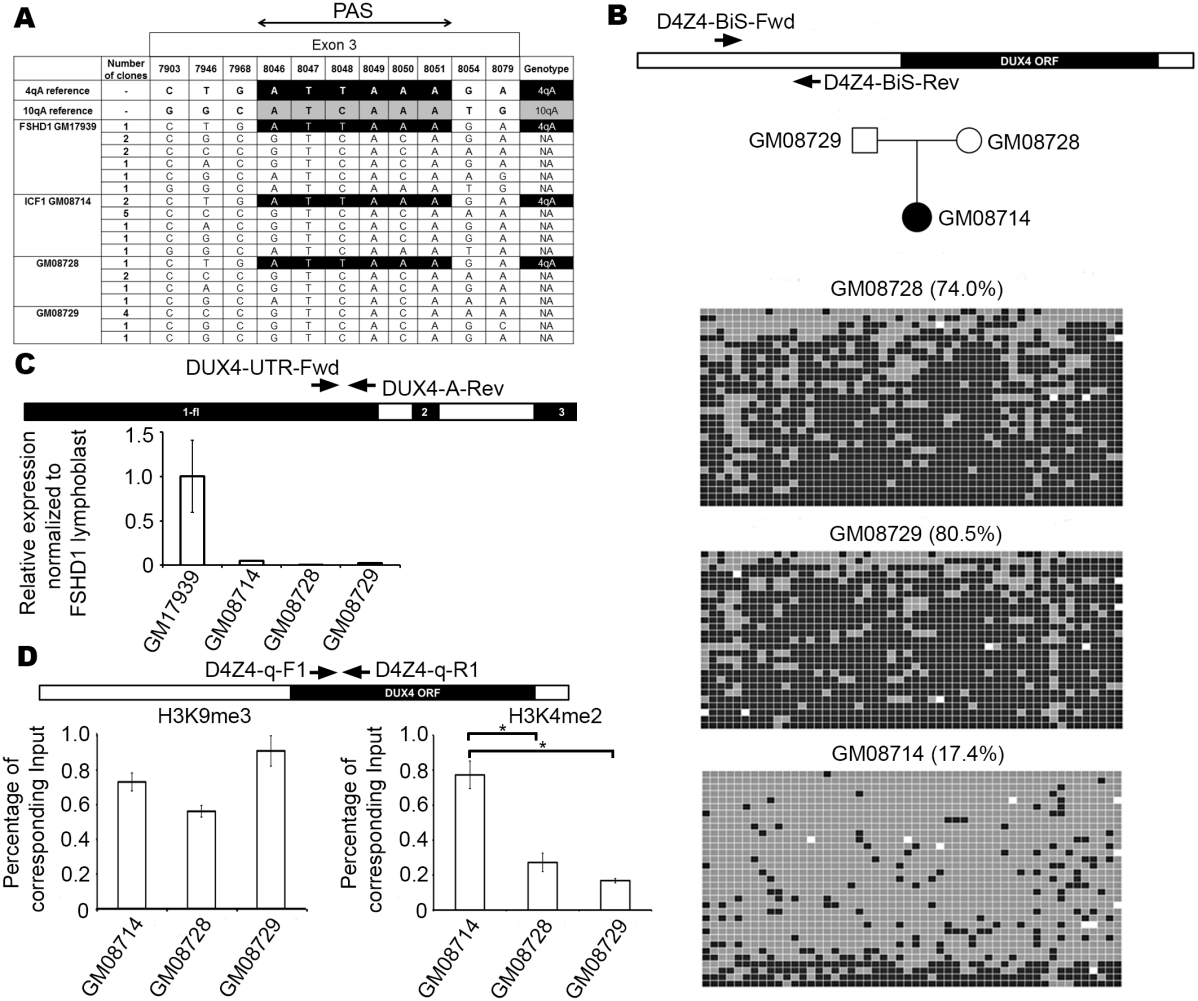
Characterization of D4Z4 in an ICF1 patient and unaffected parents. (A) Results of genotyping for ICF1 patient (GM08714) and unaffected parents (GM08728; GM08729) lymphoblastoid cell lines displayed in the same manner as described in Fig 1A. One FSHD1 patient (GM17939) lymphoblastoid cell line was genotyped as control. (B) Primer map and results of bisulfite analysis for the ICF1 patient and parent cell lines displayed in the same manner as described in Fig 1D. The family pedigree is depicted immediately below the primer map. (C) Primer map and results of qRT-PCR for D4Z4 transcription in ICF1 patient and unaffected parents, displayed in the same manner as in Fig 3C (with respect to FSHD1 lymphoblast GM17939, set arbitrarily at 1). (D) Primer map and results of qPCR for ICF1 patient and unaffected parents with ChIP using anti-H3K9me3 (left) or anti-H3K4me2 (right), displayed in the same manner as in Fig 2D. Statistical significance is indicated (* indicates p = < 0.01).

DKO cells show *DUX4-fl* transcription as well as CpG hypomethylation and a shift toward euchromatic histone methylation patterns. However, whether transcript level changes are linked to reduced DNA methylation alone, reduced histone methylation alone or require change in both, is unknown. Patients with ICF1 syndrome (OMIM # 242860) exhibit D4Z4 hypomethylation [51] due to autosomal recessive mutations in *DNMT3B* [52–54]. If CpG hypomethylation alone is sufficient to induce *DUX4* expression, an ICF1 patient with a ‘ATTAAA’ containing 4qA chromosome could potentially express *DUX4-fl* and provide an additional means to investigate the relationship between histone/DNA methylation and D4Z4 transcript levels. Initially, we genotyped distal D4Z4 in an ICF1 patient (GM08714) and her unaffected parents (Mother: GM08728; Father: GM08729). As a positive control, we included the FSHD1 patient lymphoblastoid cell line GM17939, who as anticipated, carries a pathogenic 4qA poly-A signal. The ICF1 patient also has a poly-A signal that appears to have been inherited from her mother, since we could not detect it in the father (Fig 5A). This has recently been validated in a separate study, while this manuscript was under revision [55] that reported the presence of a 4qA-L allele in this patient. We performed D4Z4 BiS analysis to confirm D4Z4 hypomethylation in the ICF1 patient (parents included as controls). In agreement with previous observations [51], we found severe and significant D4Z4 hypomethylation in the ICF1 patient (17.4%) relative to her parents (Mother: 74.0%; Father: 80.5%, p = <0.001; Fig 5B). Therefore, given the presence of a poly-A signal and CpG-hypomethylation, it is conceivable that *DUX4-fl* could be expressed in this individual. We were unable to detect *DUX4-fl* expression by nested RT-PCR with primers used in Fig 3A or with qRT-PCR Exon 2–3 primers used in Fig 3D (data not shown), possibly due to inability of our primers to detect the 4qA-L allele, similar to a recent study [55] and we could only detect very low levels of general D4Z4 transcripts in the patient by qRT-PCR (Fig 5C) using primers that amplify the 3’ region of *DUX4* ORF (Exon 1) as used in Fig 3C. If DUX4 protein is indeed expressed in the ICF1 patient avoids detection due to the limitations mentioned above, it should upregulate its target genes. Hence, we quantified transcription of *TRIM43* and *MBD3L2*. We found that both these genes are upregulated in the patient as compared to unaffected controls (S3 Fig), indicating that DUX4 protein may be expressed in the ICF1 patient but not in the mother who has a 4qA allele but does not possess a hypomethylated D4Z4. To check if histone H3 methylation levels were affected, we performed H3K9me3 and H3K4me2 ChIP on chromatin extracted from the ICF1 family cell lines and assessed the samples by qPCR. We found that compared to unaffected parents (controls), H3K9me3 enrichment was not significantly different (Fig 5D; left panel), whereas H3K4me2 was significantly higher in the patient (Fig 5D; right panel), consistent with possible activation of DUX4.

### H3K9me3 reduction alone is sufficient for D4Z4/*DUX4-fl* transcription in HCT116 and 3BKO in the absence of CpG methylation change

**Fig 6:**
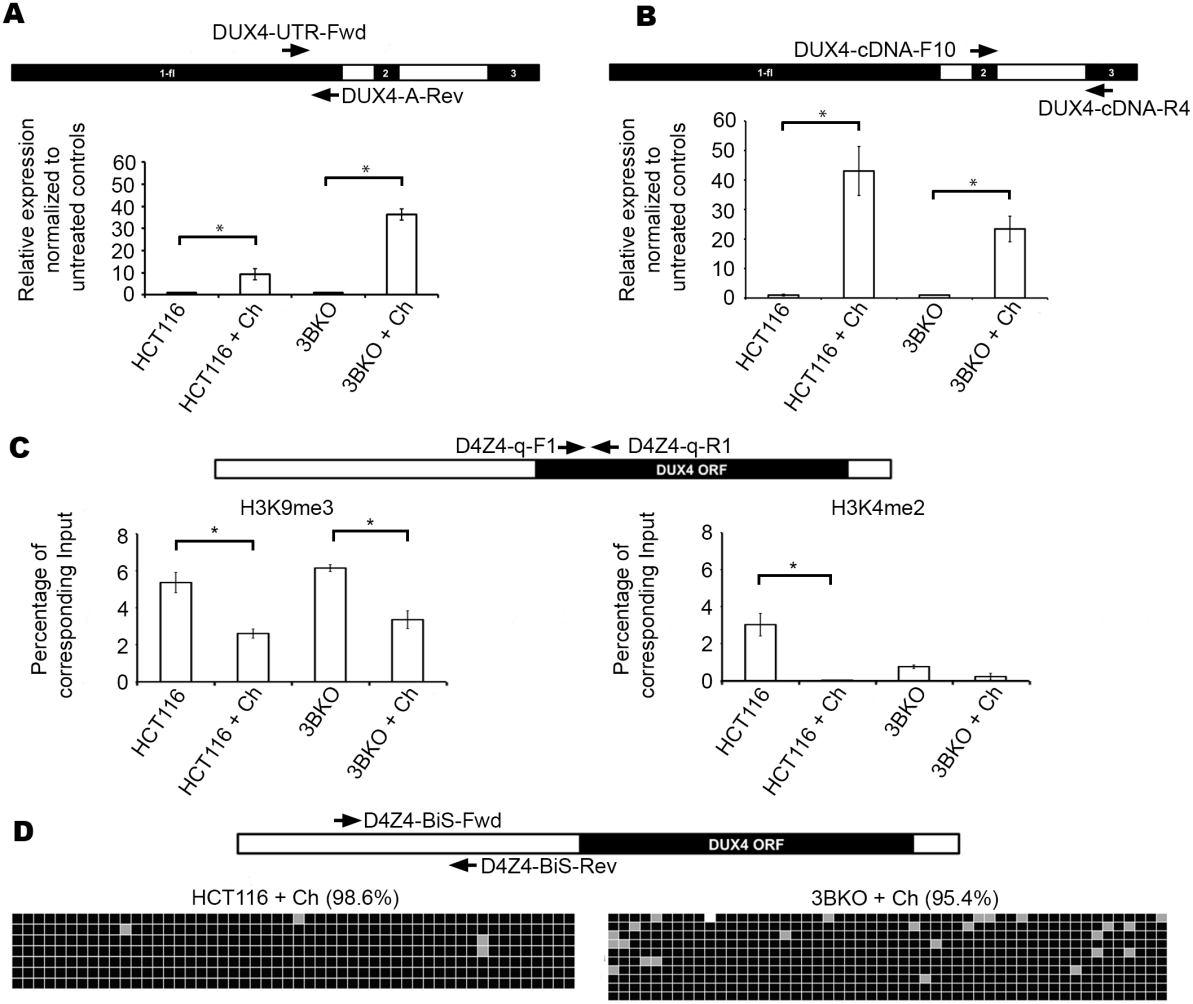
Impact of chaetocin treatment on D4Z4 in HCT116 and 3BKO cells. (A) Primer map and results of qRT-PCR for D4Z4 transcription in untreated and chaetocin treated HCT116 and 3BKO, displayed in the same manner as in Fig 3C (with respect to untreated HCT116 and 3BKO, set arbitrarily at 1). Statistical significance is indicated (* indicates p = < 0.05 for HCT116 and p = <0.001 for 3BKO). (B) Primer map and results of qRT-PCR for *DUX4-fl* in untreated and chaetocin treated HCT116 and 3BKO, displayed in the same manner as in Fig 3C (with respect to untreated HCT116 and 3BKO, set arbitrarily at 1). Statistical significance is indicated (* indicates p = < 0.05). (C) Primer map and results of qPCR for untreated and chaetocin treated HCT116 and 3BKO with ChIP using anti-H3K9me3 (left) or anti-H3K4me2 (right), displayed in the same manner as in Fig 2D. Statistical significance is indicated (* indicates p = <0.01 for left panel and p = <0.05 for right panel). (D) Primer map and results of bisulfite analysis for chaetocin treated HCT116 and 3BKO displayed in the same manner as described in Fig 1D.

The data from the ICF1 patient suggests CpG hypomethylation may have an impact on levels of D4Z4 transcripts in the presence of H3K9me3 retention. To test if H3K9me3 reduction alone can result in D4Z4/*DUX4-fl* transcription, we treated HCT116, 1KO and 3BKO cells with chaetocin. Chaetocin is a small molecule that was reported to be a specific inhibitor of the H3-K9 HMTase Suppressor of variegation 3–9 homolog 1 (SUV39H1) [56], the HMTase responsible for H3K9me3 at D4Z4 [57]. 1KO cells consistently did not survive the chaetocin treatment, hence results are depicted only for HCT116 and 3BKO. In both HCT116 and 3BKO, treatment resulted in a significant increase in D4Z4 transcription (Fig 6A) as well as that of the *DUX4-fl* transcript (Fig 6B), compared to their untreated controls. To investigate whether this D4Z4 reactivation was associated with changes to histone H3 or DNA CpG methylation, we first performed qChIP on chaetocin treated cells of HCT116 and 3BKO. We observed a significant decrease in H3K9me3 levels in both chaetocin treated HCT116 and 3BKO (Fig 6C; left panel), compared to their untreated controls. Interestingly, we observed a decrease in H3K4me2 levels in treated cells (Fig 6C; right panel), despite increased *D4Z4/DUX4-fl* transcript levels. D4Z4 BiS analysis in both HCT116 and 3BKO, revealed no significant reduction in DNA methylation in chaetocin treated cells compared to respective controls (Fig 6D).

Upon chaetocin treatment, *MYOD1* was only upregulated in 3BKO but not in parental HCT116 cells (S2B Fig). On the other hand, *MYH2* was upregulated in both chaetocin treated HCT116 and 3BKO (S2B Fig); however transcript levels were greater in 3BKO.

### D4Z4 transcription is a feature of other normal human tissues in addition to testis

**Fig 7:**
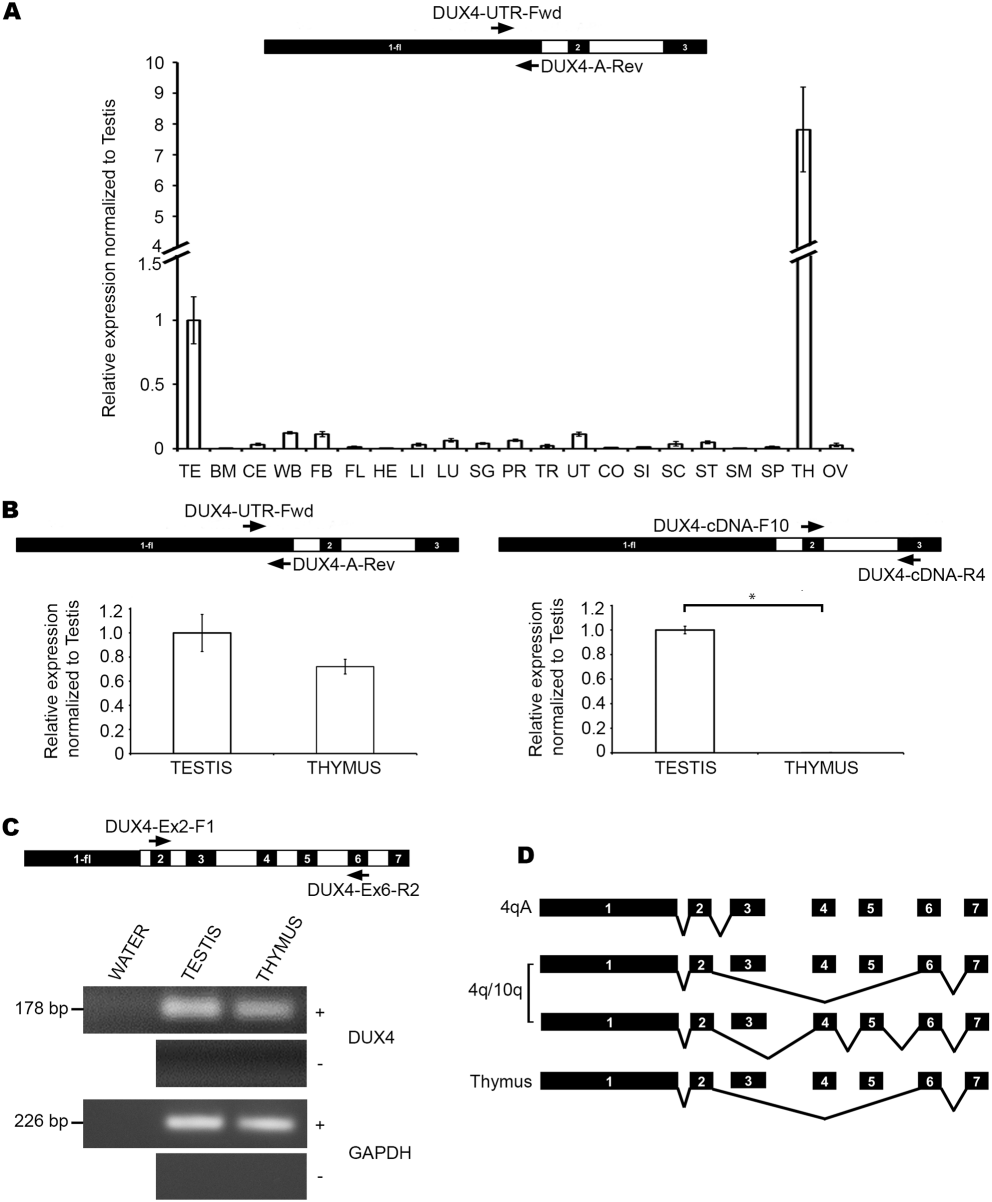
D4Z4 transcription is a feature of normal tissues other than testis. (A) Primer map and results of qRT-PCR for D4Z4 transcription for tissue cDNA made with random hexamers, in human somatic tissue panel displayed in the same manner as in Fig 3C (with respect to testis expression, arbitrarily set at 1). Samples are indicated below and include testis (TE), bone marrow (BM), cerebellum (CE), whole brain (WB), fetal brain (FB), fetal liver (FL), heart (HE), liver (LI), lungs (LU), salivary gland (SG), prostate (PR), trachea (TR), uterus (UT), colon (CO), small intestine (SI), spinal cord (SC), stomach (ST), skeletal muscle (SM), spleen (SP), thymus (TH) and ovary (OV). (B) Primer map and results of qRT-PCR for D4Z4 (left) and *DUX4-fl* (right) transcription for testis and thymus tissue cDNA made with oligo-dT primers displayed in the same manner as in Fig 6A. Statistical significance is indicated (* indicates p = < 0.01). (C) Primer map and results of RT-PCR showing expression of non-pathogenic polyadenylated mRNA transcripts in testis and thymus, displayed in the same manner as in Fig 4C. (D) Schematic diagram showing reported alternatively spliced DUX4 transcripts arising from 4q exclusively (first transcript from top), either 4q or 10q (second and third transcripts) and exon 1-2-6-7 transcript in thymus (last transcript at the bottom).

Given that we could detect very low levels of D4Z4 transcription in HCT116 cells, we questioned if this was a general feature of this cell line, or if this could be detected in normal human tissue. We first performed qRT-PCR with the exon 1 primers on cDNA prepared from total RNA extracted from tissue samples from a panel of 21 different human tissues, using random hexamers. For most of the RNAs, the tissue samples were pools from multiple individuals, for which identifying information is limited and protected (See S1 Table). We found that very low levels of D4Z4 transcription could be detected in most tissues including colon (Fig 7A). This probably indicates that D4Z4 transcription is very low in tissues of colonic origin as also seen in HCT116, in spite of its oncogenic transformation. As expected, high expression was seen in testis. However, levels of transcription were found to be highest in thymus (~8-fold higher than testis, Fig 7A). Since we do not have specific information on whether any of these tissue types possess a permissive 4qA chromosome, we did not use the exon-2/exon-3 primer set for this initial quantification. However, to further confirm the nature of the transcripts being expressed in testis and thymus, we repeated the D4Z4 and *DUX4-fl* qRT-PCRs with polyadenylated mRNA by making cDNA using oligo-dT primers. This showed that D4Z4 transcripts are expressed at levels comparable to testis, in thymus (Fig 7B-left panel). The difference in expression levels in thymus between Fig 7A and 7B (left) may be due to the different types of primers used for cDNA generation. While random priming can pick up both non-polyadenylated and poly-adenylated RNA, oligo-dT primers will selectively amplify bonafide polyadenylated transcripts arising from the array, whose expression levels are possibly lower. qRT-PCR for *DUX4-fl* expression however, showed negligible expression in thymus when compared to testis (Fig 7B-right), indicating a lack of polyadenylated transcripts consisting of 4qA exon 3. We next probed the nature of these thymus transcripts. We thus performed RT-PCR on the testis and thymus samples using forward and reverse primers in exons 2 and 6, respectively as we had done previously in HCT116 and DKO (Fig 7C). Additionally, we performed RT-PCR on these samples with the same Exon 2 forward primer and a reverse primer in exon 7 (data not shown). In both instances we found expression of transcripts of the expected size. The identity of the transcript arising from thymus was the same as that from testis for both RT-PCRs, as indicated by sequencing results (Accession numbers KX467569 and KX467570). Further analysis revealed that DUX4 target genes are also expressed in thymus (S4 Fig), albeit at lower levels than in testis, indicating the possibility of DUX4 protein expression derived from exon 7 polyadenylated transcripts in thymus. Taken together, these data indicate that D4Z4 transcription is not just restricted to the germline.

## Discussion

We sought to determine the contribution of repressive histone and DNA methylation in transcriptional silencing of the D4Z4 MSR in a context outside of FSHD. Pursuing this avenue of investigation required the identification of a cellular platform that possesses the permissive 4qA haplotype, but normally maintains D4Z4 in a transcriptionally silent state. We found that the epithelial colon carcinoma cell line HCT116 has at least one 4qA allele carrying the polyadenylation signature necessary for stabilizing *DUX4-fl*.

D4Z4 is arranged into constitutive heterochromatin in HCT116 cells as defined by DNA CpG methylation and H3K9me3. Consequently, very low levels of D4Z4-derived transcripts could be detected in these cells. However, disruption of the DNA methyltransferase genes *DNMT1* and *DNMT3B* (but not either gene alone) resulted in readily detectable polyadenylated transcripts from the array, coupled with significant reductions in both CpG methylation and H3K9me3, as well as increased levels in H3K4me2. 3BKO has shown appreciable CpG demethylation at D4Z4 previously [58]. However, the demethylation was in a region that was downstream (mid-D4Z4) of the region we have analyzed (5’ end of D4Z4) in our current study using bisulfite analysis. It is quite possible that we would have found the similar demethylation if we surveyed this downstream (mid-D4Z4) region. However, only DKO is severely hypomethylated in the 5’ end region we surveyed and expresses high levels of D4Z4-derived transcripts and not 3BKO or 1KO, which are highly methylated in this region. Thus it seems that targeted methylation by DNMT3B at sequences downstream (mid-D4Z4 and 3’-D4Z4) may not play a crucial role in *DUX4* expression. It should be noted that a recent study has suggested that DNMT3B is a modifier of DUX4 expression [55].

Genes that are bonafide targets of DUX4 protein are upregulated in DKO compared of HCT116, indicating presence of higher levels of DUX4 protein in DKO but not in HCT116. Detection of transcripts containing exons 1-2-6-7 in these cells also suggests that these germline-like transcripts could be contributing to protein expression in HCT116 and DKO, in addition to exon 1-2-3 transcripts, although further studies are needed to confirm this.

To further understand if maintenance of D4Z4 silencing was dependent on CpG methylation, H3K9me3 or both, we took two lines of investigation. We analyzed D4Z4 chromatin and transcription in an ICF1 patient that carries a permissive 4qA chromosome and hypomethylated D4Z4 but does not express *DUX4-fl*. Additionally, we pursued chemical inhibition of histone methyltransferase activity in HCT116 and its single DNMT KOs. In the ICF1 patient, despite significant reduction in D4Z4 CpG methylation and an increase in H3K4me2, H3K9me3 levels were not obviously impacted, supporting previous results [57]. Previous studies had reported that ICF1 patients do not express *DUX4*, or exhibit FSHD-like symptoms [59], but whether those patients possessed a non-canonical poly-A on a 4qA permissive chromosome was not determined. However, recently it was shown that DUX4 protein can be detected in a very small percentage of induced myotubes, along with upregulation of DUX4 target gene expression [55]. We did not study protein expression in the ICF1 patient and the lack of *DUX4-fl* transcripts that splice between exons 2 and 3 in our RT-PCR assay might be due to the inability of the primers to amplify 4qA-L transcripts [55]. However, transcripts containing the likely full ORF were detected at low levels (compared to an FSHD1 control lymphoblastoid cell line) in the ICF1 patient, agreeing with protein expression data from the abovementioned study. Additionally, DUX4 target genes were found to be upregulated in the ICF1 patient but not in her parents, indicating presence of DUX4 protein.

Surprisingly, while *DNMT3B* KO of HCT116 does not appreciably reduce CpG methylation at D4Z4, homozygous *DNMT3B* mutations in ICF1 severely hypomethylates the same region. This difference may be due to the nature of changes affecting the *DNMT3B* gene in each case. It is known that *DNMT1* and *DNMT3B* cooperatively maintain genomic methylation in HCT116 [40]. When these cells lose DNMT3B in 3BKO, DNMT1 can still be recruited and maintain methylation of some regions, such as D4Z4, which represents a bonafide target of DNMT3B. On the other hand, in ICF1 patient cells, mutations in DNMT3B C-terminal catalytic domain significantly reduce enzymatic activity [52] but possibly do not affect the ability of the mutant protein to be recruited to its target regions. This could subsequently make it difficult for other methyltransferases such as DNMT1 to methylate such regions, resulting in retention of hypomethylation. It is also possible that this difference is due to cell-type specific differences between HCT116, which is a cancerous epithelial cell line and the ICF1 patient cell line that are EBV-transformed lymphoblastoid cells. Nevertheless, upregulation of DUX4 targets in ICF1 patient in spite of very low levels of D4Z4 transcription, despite extensive CpG hypomethylation and increased H3K4me2 in the patient in this study suggested that CpG hypomethylation may be sufficient for DUX4 protein expression. This is in spite of retention of H3K9me3, which has been shown to be a central player in D4Z4 silencing [57, 60], regardless of acquisition of euchromatic features in the patient.

Chemical inhibition of SUV39H1 HMTase activity by chaetocin has previously been shown to increase *DUX4-fl* expression in normal 4qA myoblasts [60]. However, it was not clear if CpG methylation at D4Z4 was affected by such treatment. Our results in HCT116 and 3BKO with similar chaetocin treatment resulted in a significant reduction in H3K9me3 at D4Z4 and detectable *DUX4-fl*, supporting previous observations. More importantly, we found that *DUX4* expression occurred in these treated cell lines despite retention of CpG-methylation, which was a novel observation. Based on this observation, it also seems that if H3K9me3 levels are reduced, D4Z4 can be transcribed from cells irrespective of their CpG methylation status. This can be verified further using single-cell analysis. Notably, chaetocin treatment also reduced H3K4me2 levels at D4Z4, supporting observations by others that its inhibitory activity extends to other HMTases [61], although indirect effects cannot be ruled out. Nevertheless, these data further indicate that elevated H3K4me2 is not a driving factor in reactivation of D4Z4.

One important effect of DNMT loss and HMTase inhibition in HCT116 and its KOs was the upregulation of genes involved in myogenic pathways. We probed *MYOD1*, which encodes a transcription factor and is a master regulator of muscle differentiation and regeneration [62] and *MYH2*, a marker of myosin heavy chain fibers [63] to see if their expression levels changed, since *MYOD1* expression is regulated in a DNA methylation-dependent manner [64] and demethylation is known to induce myogenicity in cell types such as fibroblasts [47]. Indeed, upregulation of *MYOD1* in DKO and in chaetocin treated 3BKO but not in parental HCT116, 1KO or 3BKO indicates that loss of DNMT3B-mediated DNA methylation and not HMTase inhibition (histone modification changes), might be important for such upregulation in these cells. One question would be whether DUX4 upregulation is due to increased MYOD1 or H3K9me3 loss, as seen in DKO. MYOD1 expression level does not differ between untreated and chaetocin-treated HCT116, although DUX4 is upregulated in the latter. This suggests that DUX4 upregulation is an effect of H3K9me3 loss and not MYOD1. Nevertheless, this might be addressed either by performing *MYOD1* knockdown in DKO and monitor DUX4 expression or overexpressing MYOD1 in HCT116 cells and monitor if this induces DUX4 in the absence to H3K9me3 change.

Upregulation of *MYH2* in DKO and both chaetocin treated HCT116 and 3BKO seem to suggest that HMTase inhibition is sufficient for its expression. However, since *MYH2* levels almost doubled in chaetocin treated 3BKO as compared to chaetocin treated HCT116, it seems that similar to *MYOD1*, loss of DNMT3B-mediated DNA methylation further boosts *MYH2* transcription in presence of HMTase inhibition. It might be useful to probe the DNA methylation and histone modification status at regulatory regions of these muscle-specific genes in HCT116 and the DNMT KOs. If the epigenetic signatures correlate with expression of these genes, it will be interesting to explore how DNMT3B regulates expression these myogenic genes. Additionally, resolving the role of specific histone modification(s) in *MYH2* regulation may provide additional insights into commitment to cellular myogenic programs.

It needs to be mentioned that there are some caveats in using transformed cells such as HCT116 as opposed to primary cells or tissues. Although these cells are described as ‘near-diploid’, they might display a different modal number in some metaphases chromosomes and also a low percentage of polyploidy, along with chromosomal translocations [33–35], which makes it a deviation from a normal cell with a perfectly diploid karyotype. Also, it is reasonable to expect that results presented in this study may not translate to skeletal muscle or the FSHD phenotype, due to cell-type specific differences and regulatory factors [49]. Immortalized myoblasts [65] might be more suitable for such studies. In the current study, we focused on macrosatellite chromatin states outside of the disease context. As mentioned earlier, D4Z4 presented an ideal starting point and in spite of some of the abovementioned drawbacks, HCT116 was a suitable cellular platform for a number of reasons. These cells are immortal, are well established as a readily manipulatable cell line of choice for gene targeting [36], with readily available DNMT KOs [66]. Most importantly, this cell line contains the non-canonical exon 3 polyadenylation signal that helped us look at transcription from the array, focusing on chromatin states rather than disease phenotype. Taken together, this cell line allowed us to compare the effect of histone methylation changes in the presence (HCT116) and absence of one (1KO, 3BKO) or both (DKO) of these CpG methyltransferases.

Finally, we were able to detect very low levels of D4Z4 transcripts in most of the normal somatic tissues examined, as has been previously reported [26–27]. However, much higher levels of spliced and polyadenylated DUX4 transcripts were detected in the thymus and testis, indicating that D4Z4 transcription extends beyond the germline. Although determining the complete identity and chromosomal origin of D4Z4 transcripts arising in various tissues was beyond the scope of the current study, we need to speculate on the nature of transcripts arising from these tissues as a basis for future studies. Our results indicate that the testis produces full-length polyadenylated transcripts that utilize the 4qA exon 3. Any transcript in the testis that may splice into 10qA exon 3 would lack the ‘ATTAAA’ sequence and would be unstable. Additionally, it seems that testis also expresses transcripts that utilize the 10qA exon 7 poly-A signal, as sequencing results have verified. Previous studies support this notion that 10qA transcripts have a preference for exon 7 poly-A in the testis [27]. Expression patterns in the thymus raise two possibilities. The lack of exon 3 specific transcripts in thymus could be due to lack of a 4qA donor in the pooled mRNA sample. Although we do not have specific information on whether the any of donors for testis and thymus samples contained a 4qA allele or not, given that the tissues were derived from a Caucasian population, there is a chance that the tissue samples had one or more 4qA alleles. Alternatively, it is possible that these transcripts preferably use 4qA exon 7 poly-A just like the testis even if a 4qA exon 3 poly-A is present. Additionally, the thymus transcripts we detected could use either 4qA or 10qA exon 7 poly-A signal. However, it has been previously reported that unlike the testis, 10qA transcripts in somatic tissues do not prefer the exon 7 poly-A to that of exon 3 [27]. If this were true, these thymus transcripts would be 4qA exon 7 derived and this was supported by our sequencing data for such transcripts. Our observation that the DUX4 target genes we tested are expressed in the thymus, suggest expression of DUX4 protein in this tissue that possibly arise from such polyadenylated transcripts. Such expression is indeed novel and it needs to be determined why DUX4 and some of its target genes are expressed in the thymus, in addition to the testis. Since the testis is an immune-privileged tissue crucial for normal development, it is possible that a subset of genes in the testis are promiscuously expressed in the thymus, which is an important organ in determining ‘self-tolerance’ for tissue-specific antigens during early development. Such promiscuous expression of subsets of tissue-specific genes in the thymus has been reported previously [67]. Presentation of these testis-specific antigens to T-lymphocytes might help evade immune responses to these gene products, allowing for normal germline development to occur. It has also been shown that genes involved in immune-response pathways are downregulated when *DUX4-fl* is overexpressed in primary myoblasts [29]. Interestingly in mammals, the thymus and testis share a common feature, the lack of a conventional circadian rhythm for clock gene expression [68]. It has been speculated that since both tissues consist of differentiating cells, they lack a signal that initiates them into a cyclic pattern of clock gene expression, as seen with other tissues and instead exhibit a relatively constant pattern of clock gene expression [68]. It is possible that this is why DUX4 (and its targets) are ‘on’ in these two tissues but are silenced epigenetically in differentiated, somatic tissues such as muscle due to activation of conventional clock gene expression pattern in such tissues. This would explain why thymus is the only tissue apart from testis where we observed such high levels of DUX4 expression, although it should be noted that our tissue panel was not exhaustive and it is possible that other somatic tissues might express D4Z4 transcripts normally. Either way, these findings warrant further investigation and need to be taken into consideration when developing therapies aimed at silencing D4Z4 in FSHD patients, as systemic treatment may have a detrimental impact on the normal function of the thymus.

## Supporting Information

S1 Fig Alignment of DUX4-fl transcript sequences (indicated by arrows) expressed in DKO, with publicly annotated DUX4 transcripts on the UCSC genome browser. Top and bottom panels show transcripts with and without intron 1, respectively. Product sizes are indicated on top of each image. Images are screenshots adapted from output obtained by submitting query sequence using the BLAT tool at www.genome.ucsc.edu.
(PDF)

S2 Fig Changes in expression levels of *MYOD1* and *MYH2* transcripts in HCT116 and its DNMTKOs. (A) Results of qRT-PCR for *MYOD1* (left) and *MYH2* (right) transcription in HCT116, 1KO, 3BKO and DKO displayed in the same manner as in Fig 3C (with respect to HCT116, set arbitrarily at 1). (B)Results of qRT-PCR for *MYOD1* (left) and *MYH2* (right) transcription in untreated and chaetocin treated HCT116 and 3BKO, displayed in the same manner as in Fig 3C (with respect to untreated HCT116 and 3BKO, set arbitrarily at 1).
(PDF)

S3 Fig DUX4 target gene expression in ICF1 patient and unaffected parents. Results of qRT-PCR for DUX4 target genes *TRIM43* and *MBD3L2* in the ICF1 patient (GM08714) and unaffected parents (X-axis) expressed as fold change relative to expression in the ICF1 patient lymphoblastoid cell line (arbitrarily set at 1; Y-axis), normalized with respect to GAPDH expression. All values are obtained by averaging results from triplicates for each sample.
(PDF)

S4 Fig DUX4 target gene expression in thymus and testis. Results of qRT-PCR for DUX4 target genes *TRIM43* and *MBD3L2* in pooled thymus and testis samples (X-axis) expressed as fold change relative to expression in testis (arbitrarily set at 1;Y-axis), normalized with respect to GAPDH expression. All values are obtained by averaging results from triplicates for each sample.
(PDF)

S1 Table Table indicating the source of human tissue total RNA samples. (PDF)
