# Supplementary material for: Influence of Repressive Histone and DNA Methylation upon D4Z4 Transcription in Non-Myogenic Cells
Source: PLoS One. 2016 Jul 28;11(7):e0160022. doi: 10.1371/journal.pone.0160022 (PMC4965136; doi:10.1371/journal.pone.0160022)
Supplement: S1 Fig — Top and bottom panels show transcripts with and without intron 1, respectively. Product sizes are indicated on top of each image. Images are screenshots adapted from output obtained by submitting query sequence using the BLAT tool at www.genome.ucsc.edu. (PDF) [file pone.0160022.s001.pdf]

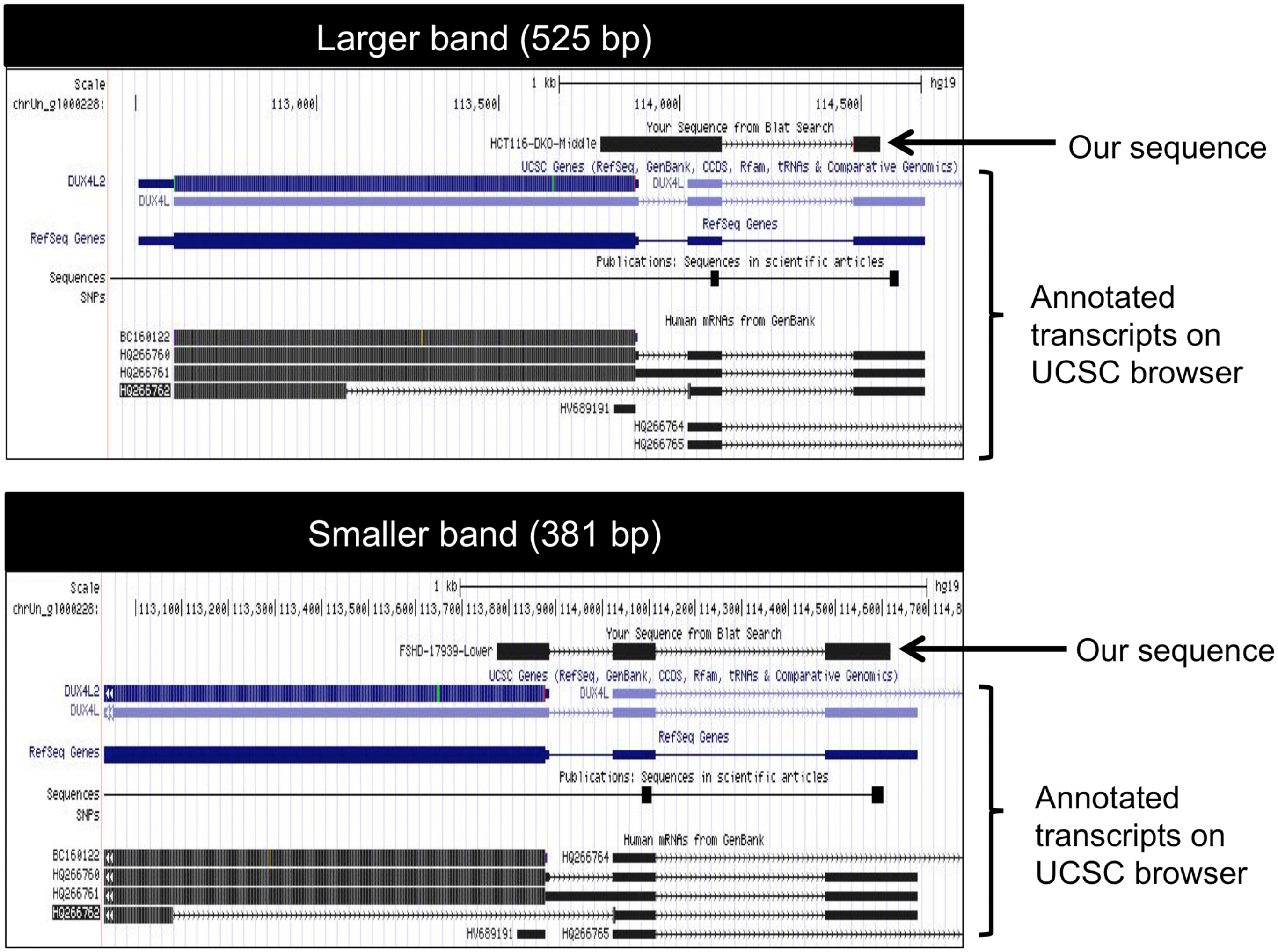

Figure legend

Supplemental Figure S1

Alignment of *DUX4-fl* transcript sequences (indicated by arrows) expressed in DKO, with publicly annotated *DUX4* transcripts on the UCSC genome browser. Top and bottom panels show transcripts with and without intron 1, respectively. Product sizes are indicated on top of each image. Images are screenshots adapted from output obtained by submitting query sequence using the BLAT tool at www.genome.ucsc.edu.
