# Supplementary material for: Influence of Repressive Histone and DNA Methylation upon D4Z4 Transcription in Non-Myogenic Cells
Source: PLoS One. 2016 Jul 28;11(7):e0160022. doi: 10.1371/journal.pone.0160022 (PMC4965136; doi:10.1371/journal.pone.0160022)
Supplement: S2 Fig — (A) Results of qRT-PCR for MYOD1 (left) and MYH2 (right) transcription in HCT116, 1KO, 3BKO and DKO displayed in the same manner as in Fig 3C (with respect to HCT116, set arbitrarily at 1). (B)Results of qRT-PCR for MYOD1 (left) and MYH2 (right) transcription in untreated and chaetocin treated HCT116 and 3BKO, displayed in the same manner as in Fig 3C (with respect to untreated HCT116 and 3BKO, set arbitrarily at 1). (PDF) [file pone.0160022.s002.pdf]

A

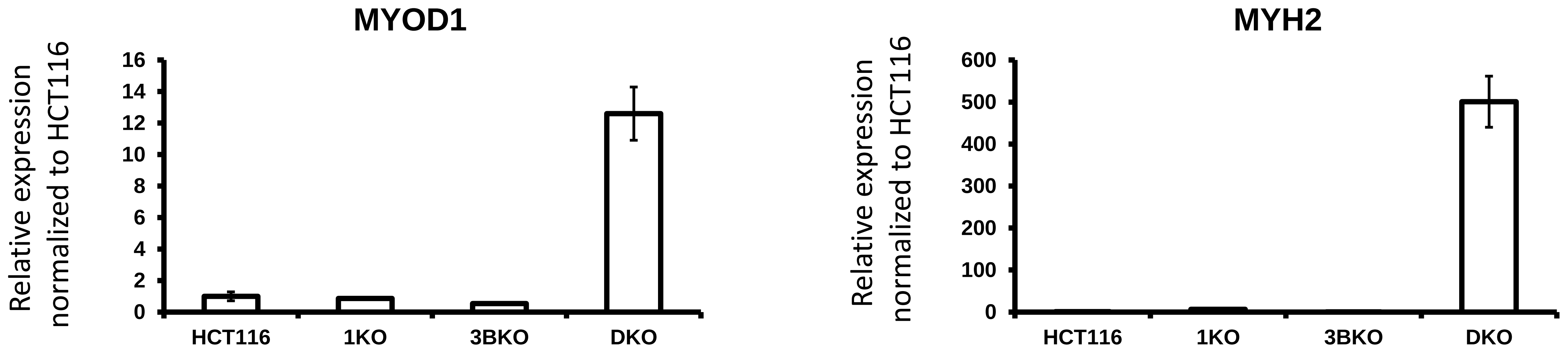
MYOD1
Relative expression normalized to HCT116
16
14
12
10
8
6
4
2
0
HCT116
1KO
3BKO
DKO
MYH2
Relative expression normalized to HCT116
600
500
400
300
200
100
0
HCT116
1KO
3BKO
DKO

B

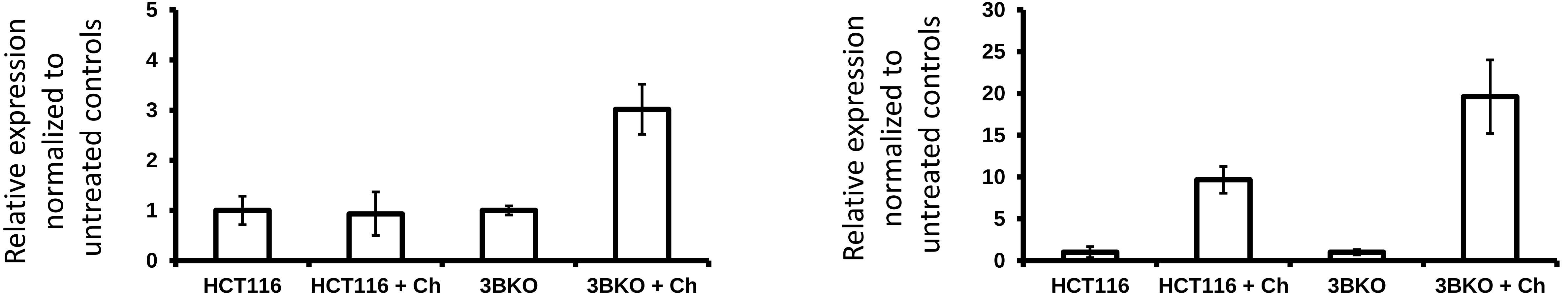
Relative expression normalized to untreated controls
5
4
3
2
1
0
HCT116
HCT116 + Ch
3BKO
3BKO + Ch
Relative expression normalized to untreated controls
30
25
20
15
10
5
0
HCT116
HCT116 + Ch
3BKO
3BKO + Ch

Figure legend

Supplemental Fig. 2. Changes in expression levels of *MYOD1* and *MYH2* transcripts in HCT116 and its DNMT KOs

(A) Results of qRT-PCR for *MYOD1* (left) and *MYH2* (right) transcription in HCT116, 1KO, 3BKO and DKO displayed in the same manner as in Fig 3C (with respect to HCT116, set arbitrarily at 1).

(B) Results of qRT-PCR for *MYOD1* (left) and *MYH2* (right) transcription in untreated and chaetocin treated HCT116 and 3BKO, displayed in the same manner as in Fig 3C (with respect to untreated HCT116 and 3BKO, set arbitrarily at 1).

Primers used for qRT-PCR of myogenic genes (5' to 3')
MYOD1
qMYOD1-F1: TCGAAACACGGGTCGTCAT
qMYOD1-R1: CGGCGGAACTGCTACGAAG

MYH2
qMYH2-F1: GGACCAACTGAGTGAACTGAAA
qMYH2-R1: TTGCCTCTTGATAACTGAGACAC
