# Supplementary material for: Influence of Repressive Histone and DNA Methylation upon D4Z4 Transcription in Non-Myogenic Cells
Source: PLoS One. 2016 Jul 28;11(7):e0160022. doi: 10.1371/journal.pone.0160022 (PMC4965136; doi:10.1371/journal.pone.0160022)
Supplement: S3 Fig — Results of qRT-PCR for DUX4 target genes TRIM43 and MBD3L2 in the ICF1 patient (GM08714) and unaffected parents (X-axis) expressed as fold change relative to expression in the ICF1 patient lymphoblastoid cell line (arbitrarily set at 1; Y-axis), normalized with respect to GAPDH expression. All values are obtained by averaging results from triplicates for each sample. (PDF) [file pone.0160022.s003.pdf]

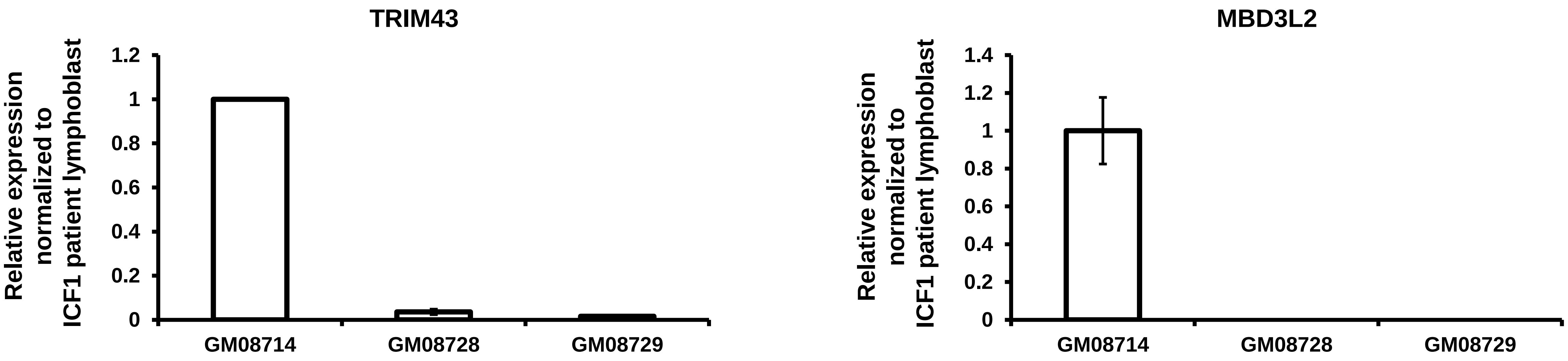

Figure legend

Supplemental Figure S3. DUX4 target gene expression in ICF1 patient and unaffected parents

Results of qRT-PCR for DUX4 target genes *TRIM43* and *MBD3L2* in the ICF1 patient (GM08714) and unaffected parents (X-axis) expressed as fold change relative to expression in the ICF1 patient lymphoblastoid cell line (arbitrarily set at 1; Y-axis), normalized with respect to GAPDH expression. All values are obtained by averaging results from triplicates for each sample.
