# Supplementary material for: Influence of Repressive Histone and DNA Methylation upon D4Z4 Transcription in Non-Myogenic Cells
Source: PLoS One. 2016 Jul 28;11(7):e0160022. doi: 10.1371/journal.pone.0160022 (PMC4965136; doi:10.1371/journal.pone.0160022)
Supplement: S1 Table — (PDF) [file pone.0160022.s005.pdf]

Supplementary Table 1: Tissue Total RNA Samples

Product: Human Total RNA Master Panel II/Clontech (Cat. No. 636643, Lot Number, 1001243A).

Tissue	Source
Bone Marrow	Pooled from 3 male Asians, ages: 56-67
Brain, cerebellum	Pooled from 10 male/female Caucasians, ages: 22-68
Brain (whole)	18-year-old male Caucasian
Fetal brain	Pooled from 59 spontaneously aborted male/female Caucasian fetuses, age: 20-33 weeks
Fetal liver	Pooled from 63 spontaneously aborted male/female Caucasian fetuses, ages: 22-40 weeks
Heart	Pooled from 3 male Caucasians, ages: 30-39
Liver	51-year-old male Caucasian
Lung	Pooled from 3 male/female Caucasians, ages: 32-61
Prostate	Pooled from 12 Caucasians, ages: 20-58
Salivary gland	Pooled from 24 male/female Caucasians, ages: 16-60
Skeletal muscle	Pooled from 2 male/female Caucasians, ages: 43-46
Spleen	Pooled from 12 male/female Caucasians, ages: 18-54
Testis	Pooled from 39 Caucasians, ages: 14-64
Thymus	Pooled from 2 male Caucasians, ages: 18-57
Trachea	Pooled from 22 male/female Caucasians, ages: 18-54
Uterus	Pooled from 8 Caucasians, ages: 23-63
Colon w/mucosa	23-year-old female Caucasian
Small intestine	Pooled from 5 male/female Caucasians, ages: 20-61
Spinal cord	Pooled from 22 male/female Caucasians, ages: 20-69
Stomach	50-year-old male Caucasian
Ovary*	47-year-old female

*Source: Agilent Technologies, Catalog Number 540071
